# EZH2-mediated loss of miR-622 determines CXCR4 activation in hepatocellular carcinoma

**DOI:** 10.1038/ncomms9494

**Published:** 2015-09-25

**Authors:** Haiou Liu, Yidong Liu, Weisi Liu, Weijuan Zhang, Jiejie Xu

**Affiliations:** 1 Shanghai Key Laboratory of Female Reproductive Endocrine Related Diseases, Hospital of Obstetrics and Gynecology, Fudan University, Shanghai 200011, China; 2 Department of Biochemistry and Molecular Biology, School of Basic Medical Sciences, Fudan University, Shanghai 200032, China; 3 Department of Immunology, School of Basic Medical Sciences, Fudan University, Shanghai 200032, China

## Abstract

The CXC chemokine receptor 4 (CXCR4) exerts a variety of functions at different steps of hepatocellular carcinoma (HCC) progression. The molecular mechanisms and therapeutic value of CXCR4 in the development of HCC remain undefined. Here we show that aberrant CXCR4 overexpression is associated with poor prognosis and aggressive characteristics of HCC. Suppression of CXCR4 activity via CXCR4 knockdown, AMD3100 or neutralizing antibody administration inhibits hepatoma cell tumorigenesis *in vitro* and *in vivo*. CXCR4 overexpression displays the opposite effects. Using Mir library screening we identify miR-622 as a regulator of CXCR4. Further studies show that miR-622 directly target the 3′ untranslated region of CXCR4 and is transcriptionally repressed by EZH2-induced H3K27 trimethylation and promoter methylation. EZH2/miR-622 promotes tumorigenesis through CXCR4. EZH2-mediated loss of miR-622 is found to correlate with CXCR4 overexpression and unfavourable prognosis in HCC patients. This study establishes EZH2/miR-622/CXCR4 as a potential adverse prognostic factor and therapeutic target for HCC patients.

## Abstract (web summary)

Chemokines and their receptors have key roles in tumorigenesis. Here, the authors demonstrate that CXRC4 is overexpressed in hepatocellular carcinoma and is associated with poor prognosis and, mechanistically CXCR4 is increased in expression via EZH2 repression of microRNA-622.

## Graphical abstract

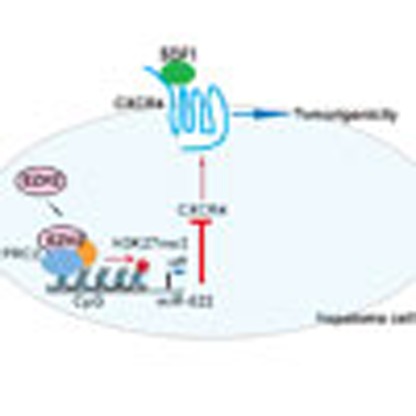

The CXC chemokine receptor 4 (CXCR4) and its chemokine ligand 12 (CXCL12) have implicated in many key steps of cancer, including angiogenesis, epithelial–mesenchymal transition, invasion, dissemination and cancer cell stemness^1^,^2^. After CXCL12 binding, CXCR4 activates different pathways, notably calcium release and cellular migration, PI3K/AKT and cellular survival, Ras-MAPK and cell proliferation, β-catenin and cancer cell stemness^3^,^4^. The CXCR4/CXCL12 has multiple functions at various points in the progression of hepatocellular carcinomas (HCCs). Both autocrine and/or paracrine effects of this pathway have been shown to maintain cancer growth, induce angiogenesis and aid escape of immune surveillance^5^. The molecular mechanisms that explain CXCL12 and CXCR4 expression in HCC remain undefined.

Numerous studies have demonstrated immunohistochemical staining of CXCR4 in HCC tissues but not in normal hepatic tissues^6^,^7^. CXCR4 mRNA expression are contrasting, Liu *et al*.^7^ found overexpression in HCC tumour tissues, while others report reduced expression in HCC tissues or no differences^6^. Nonetheless, the majority of studies showed correlations between CXCR4 expression and aggressive tumour behaviour and poor clinical outcome^6^,^8^. Understanding the regulation network of CXCR4 would give us a deeper insight into the mechanisms underlying hepatocarcinogenesis.

The microRNAs (miRNAs) constitute small non-coding RNAs (19–22 nucleotides) that provoke mRNA degradation or blockade of mRNA translation by interacting with the 3′ untranslated regions (3′-UTRs) of target mRNAs^9^. Importantly, downregulation of some miRNAs can motivate tumorigenesis through the upregulation of oncogenes and silencing of tumour suppressor genes, respectively^10^. miR-622 functions as a tumour suppressor by targeting *K-RAS*^11^. It is downregulated in human gastric carcinoma tissues, pancreatic adenocarcinoma and ampullary adenocarcinoma^12^,^13^. In the context of HCC, aberrant expression of specific miRNAs are closely associated with tumour cell proliferation, migration and invasion by targeting proteins involved in these cellular functions^14^,^15^,^16^. However, the expression and the role of miR-622 in HCC have not been clearly demonstrated.

In this work, we identify CXCR4 overexpression in a subset of HCCs, which contributing to hepatoma cell proliferation, colony formation, migration and survival. Suppression of CXCR4 activity either by shRNA or pharmacological inhibition suppresses hepatoma cell growth *in vitro* and *in vivo*. A comprehensive miRNA analysis reveals that CXCR4 expression regulated by miR-622, which is epigenetically downregulated by enhancer of zeste homologue 2 (EZH2). Moreover, EZH2/miR-622 pathway is significantly associated with CXCR4 expression and poor prognosis of HCC patients. Thus, the alteration in the EZH2/miR-622/CXCR4 pathway contributes to tumour development and represents therapeutic targets.

## Results

### CXCR4 is upregulated in HCC and correlated with survival

**Figure 1:**
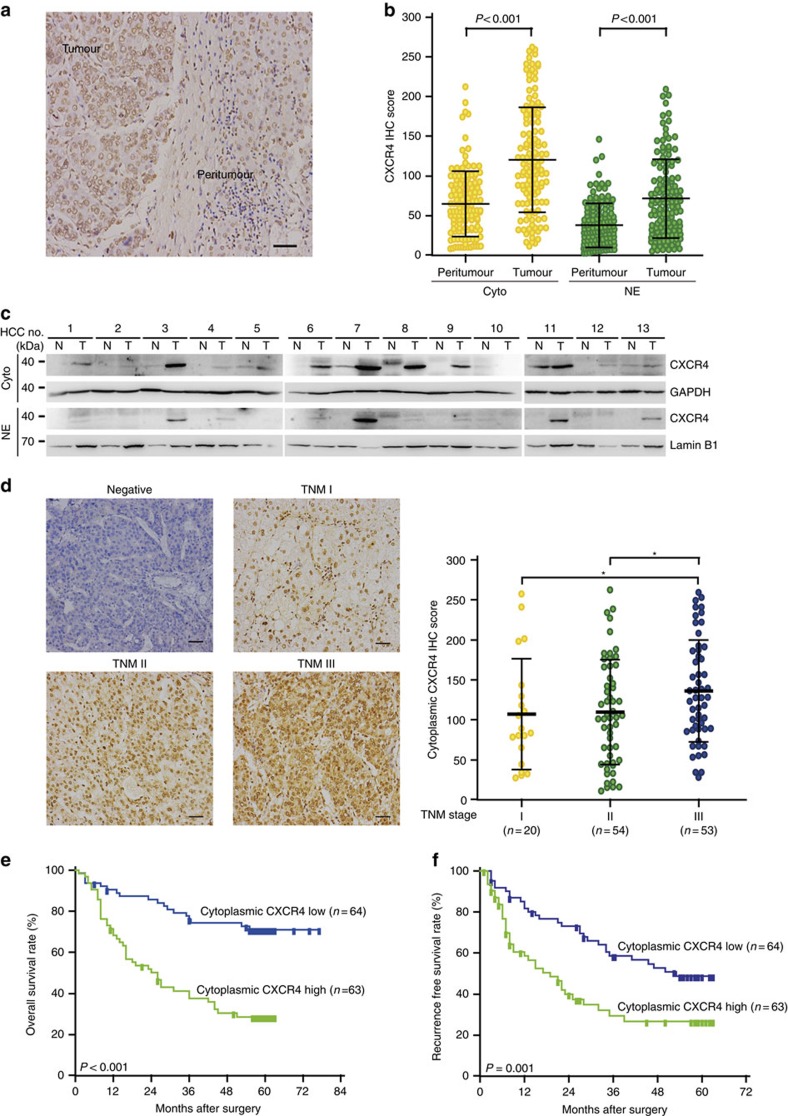
Upregulation of CXCR4 in HCC correlated with poor patient survival. (**a**) Representative immunohistochemistry (IHC) staining with CXCR4 (scale bar, 50 μm). (**b**) Scatter plots for corresponding evaluated IHC score in tumour and peritumour HCC specimens (*n*=127). The horizontal lines in the plots represent the median and the interquartile range. The *P* values were calculated using Student's *t*-test. (**c**) Western blot analysis of CXCR4, GAPDH in cytoplasmic extracts (Cyto) and CXCR4, lamin B1 in nuclear extracts (NE) in HCC specimens. Data are representative immunoblots of three independent assays. (**d**) Representative IHC staining with CXCR4 from TNM stage I to III of HCC specimens (scale bar, 50 μm)(left panel). Scatter plots for corresponding evaluated IHC score from TNM stage I to III of HCC specimens (right panel). Error bars represent mean±s.d. IHC scores were compared by one-way analysis of variance and Student's *t*-test, *P<0.05. (**e**,**f**) Kaplan–Meier plots indicate the overall survival (**e**) and RFS (**f**) for HCC patients categorized by CXCR4 expression (*n*=63 for high-CXCR4 group versus *n*=64 for low-CXCR4 group), *P* value is determined by log-rank test.

To investigate the potential significance of CXCR4 in the development and progression of HCC, we first evaluated CXCR4 expression by immunohistochemical analysis in 127 HCC specimens. CXCR4 was located diffusely in the cytoplasm and nucleus of tumour cells (Fig. 1a). However, the level of cytoplasmic and nuclear CXCR4 expressions was significantly higher in tumour than peritumour tissues (Fig. 1b). Immunoblot analysis of lysates obtained from surgical samples of 13 HCC patients confirmed increases of CXCR4 expression in tumour relative to peritumour tissues (Fig. 1c). As shown in Supplementary Table 1, the level of cytoplasmic CXCR4 expression closely correlated with tumour size (*P*=0.003, *χ*^2^-test), venous invasion (*P*=0.006, *χ*^2^-test), high Barcelona Clinic Liver Cancer stage (*P*=0.003, χ2-test) and Tumour, Node, Metastasis (TNM) stage (*P*<0.001, *χ*^2^-test). The level of cytoplasmic CXCR4 expression was correlated with tumour progression from TNM stage I to III (Fig. 1d). Importantly, patients with high cytoplasmic CXCR4 intensity showed significantly worse overall survival (mean of 33.0 versus 61.3 months, log-rank test *P*<0.001; Fig. 1e) and recurrence-free survival (RFS; mean of 26.9 versus 42.6 months, log-rank test *P*=0.001; Fig. 1f) than those with low cytoplasmic CXCR4 expression, while nuclear CXCR4 expression was found not to be significantly related to overall survival and RFS (Supplementary Fig. 1). The Cox proportional hazards model revealed that high cytoplasmic CXCR4 expression was an independent prognostic factor with respect to overall survival (hazard ratio=1.889 (95% confidence interval, 1.040–3.431), *P*=0.038) and RFS (hazard ratio=1.695 (95% confidence interval, 1.008–2.853), *P*=0.048) (Table 1 and Supplementary Table 2). Taken together, it was clearly indicated that cytoplasmic CXCR4 expression was a significant and independent index for HCC outcomes.

### CXCR4 is required for tumour growth, migration and survival

**Figure 2:**
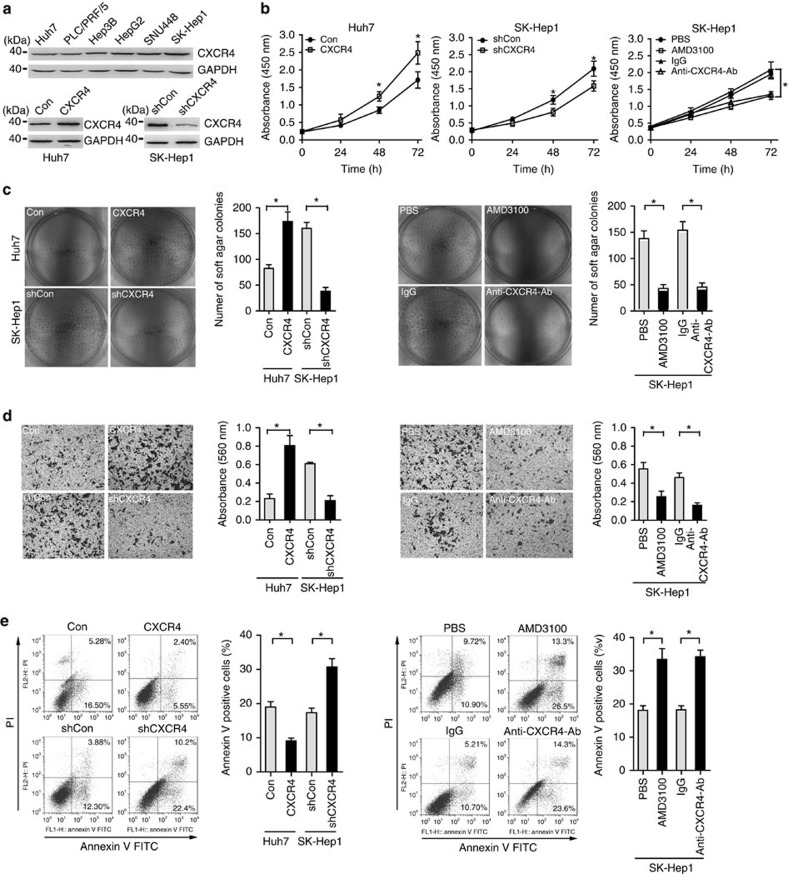
CXCR4 promotes hepatoma cell proliferation and survival *in vitro*. (**a**) Western blot analysis of CXCR4 and GAPDH for Huh7, PLC/PRF/5, Hep3B, HepG2, SNU448 and SK-Hep1 cells (upper panel), for Huh7 cells stably transfected with empty vector or CXCR4 (lower left panel) and for SK-Hep1 cells stably transfected with nonspecific shRNA (shCon) or CXCR4-specific shRNA (shCXCR4) (lower right panel). Data are representative immunoblots of three independent assays. (**b**) Cell proliferation analysis for Huh7 cells without or with stably CXCR4 overexpression, SK-Hep1 cells without or with stably CXCR4 knockdown, SK-Hep1 cells without or with AMD3100 (100 nM) or CXCR4-neutralization antibody (100 μg ml^−1^) treatment (*n*=3). Student's *t*-test, *P<0.05. Error bars in panels are defined as s.d. (**c**) Representative micrographs and quantification of soft agar colonies for above-mentioned hepatoma cells (*n*=3). Student's *t*-test, *P<0.05. Error bars in panels are defined as s.d. (**d**) Representative micrographs and quantification of the above-mentioned hepatoma cells in the Transwell migration assay (*n*=3). Student's *t*-test, *P<0.05. Error bars in panels are defined as s.d. (**e**) Representative dot plots of flow cytometric analysis of above-mentioned hepatoma cells cultured in serum-deprivation medium for 48 h. Cells were subjected to annexin-V and Propidium lodide (PI) staining, and the percentage of cells displaying annexin-V single positive and annexin-V, PI double positive are denoted (*n*=3). Student's *t*-test, *P<0.05. Error bars in panels are defined as s.d.

To explore the biologic function of CXCR4, we first determined the endogenous CXCR4 expression in hepatoma cell lines, and identified Huh7 and SK-Hep1 cells with the lowest and highest CXCR4 expression for subsequent experiments (Fig. 2a). We confirmed stable knockdown of CXCR4 in SK-Hep1 cells, and stable CXCR4-transfected Huh7 cells (Fig. 2a and Supplementary Fig. 2a). While ectopic CXCR4 expression promoted the proliferation of Huh7 cells, stably CXCR4 knockdown, AMD3100 (CXCR4 antagonist) and CXCR4-neutralization antibody inhibited the proliferation of SK-Hep1 cells (Fig. 2b). Furthermore, CXCR4 overexpression enhanced anchorage-independent growth, migration and survival of Huh7 cells, which were impaired by either CXCR4 knockdown, AMD3100 or neutralization antibody in SK-Hep1 cells (Fig. 2c–e).

### Suppression of CXCR4 inhibits tumorigenesis *in vivo*

**Figure 3:**
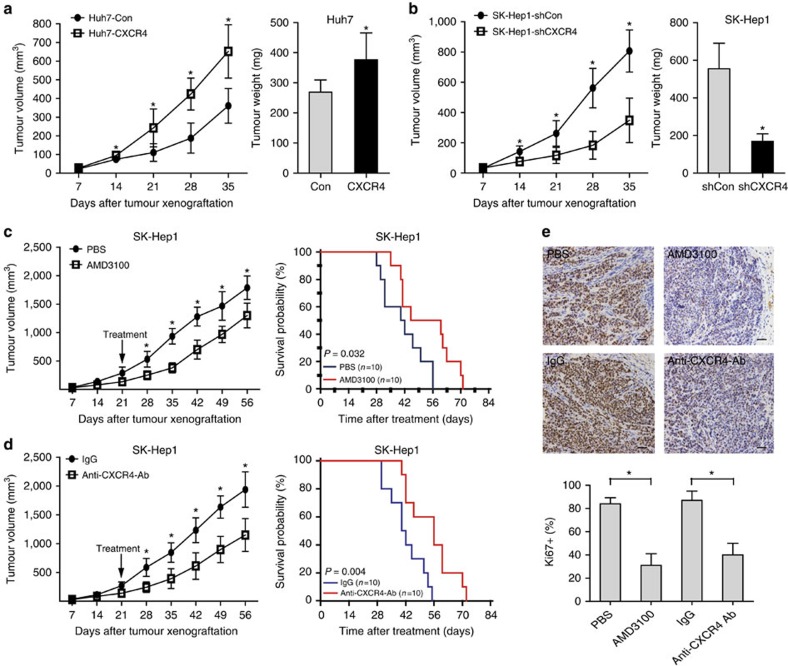
CXCR4 promotes hepatoma cells growth *in vivo*. (**a**) Tumour growth curve and quantification of weight of the tumours that developed in nude mice injected subcutaneously with Huh7-control or Huh7-CXCR4 cells (*n*=10). Student's *t*-test, *P<0.05. Error bars in panels are defined as s.d. (**b**) Tumour growth curve and quantification of weight of the tumours that developed in nude mice injected subcutaneously with SK-Hep1-shCon or SK-Hep1-shCXCR4 cells (*n*=10). Student's *t*-test, *P<0.05. Error bars in panels are defined as s.d. (**c**) Tumour growth curve of mice bearing SK-Hep1 xenografts following intratumoural administration of PBS or 10 mg kg^−1^ AMD3100 (*n*=10). Student's *t*-test, *P<0.05. Error bars in panels are defined as s.d. (left panel). Kaplan–Meier survival analysis was performed for above-mentioned xenograft mice (*n*=10), *P* value is determined by log-rank test (right panel). (**d**) Tumour growth curve analysis of mice bearing SK-Hep1 xenografts following administration of 1 mg IgG or anti-CXCR4 neutralization antibody (*n*=10). Student's *t*-test, *P<0.05. Error bars in panels are defined as s.d. (left panel). Kaplan–Meier survival analysis was performed for above-mentioned xenograft mice (*n*=10), *P* value is determined by log-rank test (right panel). (**e**) Representative IHC staining of Ki67 for SK-Hep1 xenografts with indicated administrations (scale bar, 50 μm; upper panel). Quantification of the percentage of Ki67-positive cells (*n*=10, lower panel). Student's *t*-test, *P<0.05. Error bars in panels are defined as s.d.

We next investigated the effect of CXCR4 on tumorigenesis of hepatoma cells *in vivo.* Quantification of tumour size and weight showed that Huh7 cells with CXCR4 overexpression generated larger tumours than control cells (Fig. 3a). Conversely, SK-Hep1 cells with CXCR4 knockdown generated smaller tumours than control cells (Fig. 3b). To assess the therapeutic potential of targeting CXCR4 *in vivo*, we tested subcutaneous exnografts of SK-Hep1 cells. Transplant recipient nude mice with palpable tumours were treated with AMD3100 for 3 weeks. AMD3100 treatment potently suppressed tumorigenesis (Fig. 3c, left panel). Intratumoural injection of AMD3100 had extended survival (mean of 52.7 versus 41.2 days, log-rank test *P*=0.032; Fig. 3c right panel). To further validate the role of CXCR4 in tumorigenesis, we turned to a CXCR4-neutralization antibody. CXCR4-neutralization antibody treatment suppressed tumour size, and had extended survival (mean of 54.4 versus 42.1 days, log-rank test *P*=0.004; Fig. 3d). This was accompanied by commensurate reduction in ki67 staining (Fig. 3e). These data indicate that CXCR4 promoted tumorigenesis *in vivo* and *in vitro*, and targeting CXCR4 is a potential candidate for clinical application to the treatment of HCC.

### Identification of endogenous miRNA directly target CXCR4

**Figure 4:**
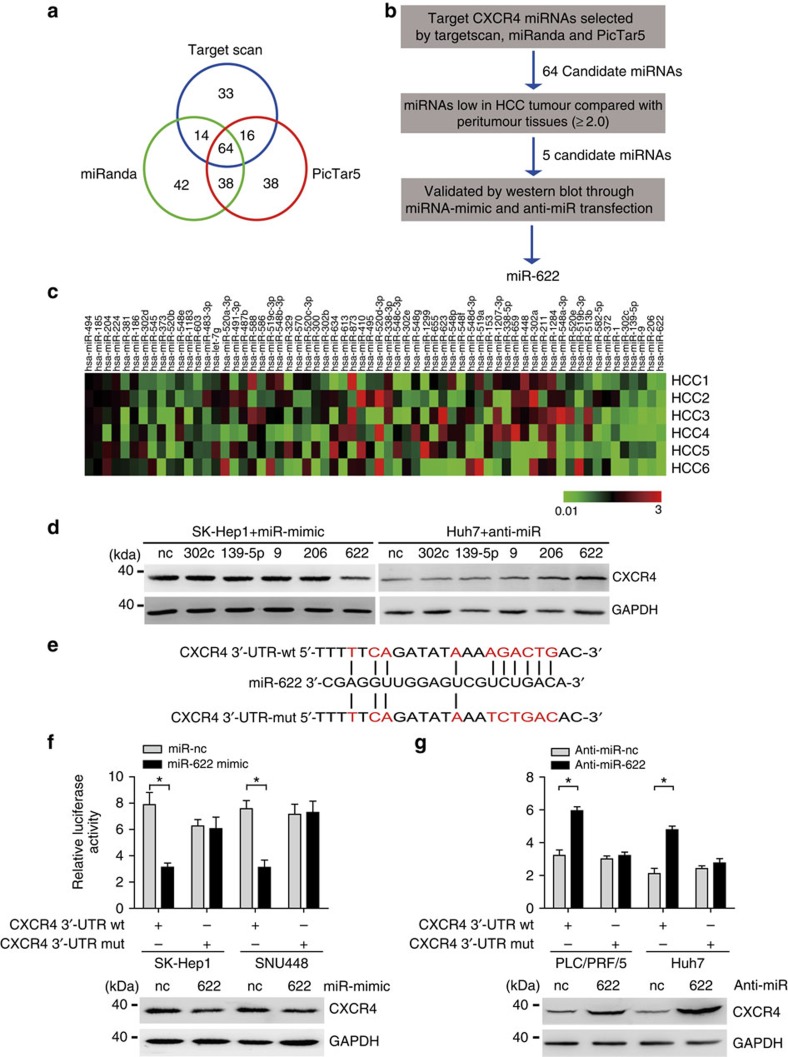
CXCR4 is a direct miR-622 target. (**a**) Schema of the candidate miRNAs by different prediction algorithms. Each labelled circle represents one prediction algorithm with the number of its predicted miRNAs, and the number listed in overlapping of circles is simultaneously predicted by different algorithms. (**b**) Schematic model for miRNA screening to target CXCR4. (**c**) Heatmap obtained from RT–PCR of HCC and corresponding peritumour specimens. Each column represents the average of three biological replicates. The relative high expression is indicated in red, whereas the relative low expression is in green. (**d**) Western blot analysis of CXCR4 and GAPDH for SK-Hep1 cells transiently transfected with miR-nc, miR-302c, miR-139-5p, miR-9, miR-206 and miR-622 mimic (left panel) and for Huh7 cells transiently transfected with anti-miR-nc, anti-miR-302c, anti-miR-139-5p, anti-miR-9, anti-miR-206 and anti-miR-622 (right panel). Data are representative immunoblots of three independent assays. (**e**) Sequences of miR-622 and the potential miR-622-binding sites at the 3′-UTR of CXCR4. Also shown are nucleotides mutated in CXCR4-3′-UTR mutant. Seed sequences are marked. (**f**) Luciferase activity assay for pGL3-CXCR4 3′-UTR (wt) or pGL3-CXCR4 3′-UTR (mut) relative to Renilla luciferase activity for SK-Hep1 and SNU448 cells transiently transfected with miR-nc or miR-622-mimc (*n*=3, upper panel). Student's *t*-test, *P<0.05. Error bars in panels are defined as s.d. Western blot analysis of CXCR4 and GAPDH for SK-Hep1 and SNU448 cells transiently transfected with miR-nc and miR-622-mimc (lower panel). Data are representative immunoblots of three independent assays. (**g**) Luciferase activity assay for pGL3-CXCR4 3′-UTR (wt) or pGL3-CXCR4 3′-UTR (mut) relative to Renilla luciferase activity for PLC/PRF/5 and Huh7 cells transiently transfected with anti-miR-nc or anti-miR-622 (*n*=3, upper panel). Student's *t*-test, *P<0.05. Error bars in panels are defined as s.d. Western blot analysis of CXCR4 and GAPDH for PLC/PRF/5 and Huh7 cells transiently transfected with anti-miR-nc or anti-miR-622 (lower panel). Data are representative immunoblots of three independent assays.

Changes in the expression of miRNAs appear to be a common characteristic of cancers including HCC^17^. Loss or suppression of miRNAs targeting CXCR4 may cause aberrant overexpression of CXCR4 in HCC. Therefore, we used comprehensive bioinformatics analysis as a filter to generate a selective miRNA library for subsequent screening. A total 64 miRs were successfully identified as candidate miRs (Fig. 4a and Supplementary Fig. 3). Screening with the candidate miR library was carried out by quantitative PCR for the downregulation of miRs in HCC samples compared with peritumour tissues (Fig. 4b). We found five miRNAs (miR-302c, miR-139-5p, miR-9, miR-206 and miR-622) were downregulated in HCC compared with peritumour tissues (Fig. 4c and Supplementary Table 3). To determine whether CXCR4 expression is selectively regulated by the five aforementioned miRs, we transfected these selected miR-mimics or anti-miRs in hepatoma cells. We found that miR-622 mimic suppressed CXCR4 expression in SK-Hep1 cells, whereas anti-miR-622 increased CXCR4 expression in Huh7 cells, which suggested that miR-622 is a specific regulator of CXCR4 in hepatoma cells (Fig. 4d). The TargetScan algorithm showed that the bases from 71 to 77 in the *CXCR4* 3′-UTR have perfect complementarity to the seed sequence of miR-622. To substantiate the site-specific repression of miR-622 on *CXCR4*, we constructed a mutated *CXCR4* 3′-UTR luciferase reporter (Fig. 4e), which completely restored luciferase activity induced by miR-622 mimic (Fig. 4f), and suppressed luciferase activity induced by anti-miR-622 (Fig. 4g). These data suggest that CXCR4 is a novel direct target of miR-622 in hepatoma cells.

### CXCR4 mediate the effects of miR-622 on tumour promotion

**Figure 5:**
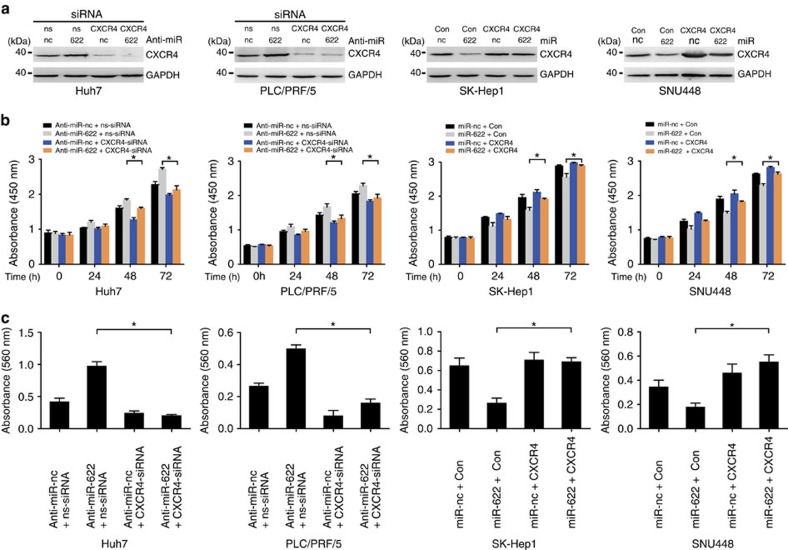
CXCR4 mediate the effects of miR-622 on hepatoma cell growth and migration. Hepatoma cells were transfected with anti-miR-622 or miR-622 before transfection with either CXCR4-siRNA or CXCR4. (**a**) Immunoblot analysis showing the inhibition of anti-miR-622-induced upregulation of CXCR4 by CXCR4-siRNA in Huh7 and PLC/PRF/5 cells, and showing the rescue of miR-622-induced downregulation of CXCR4 by CXCR4 in SK-Hep1 and SNU448 cells. Data are representative immunoblots of three independent assays. (**b**) Growth assay showing that CXCR4 inhibition partially reverse the proliferative effects of anti-miR-622 promotion in Huh7 and PLC/PRF/5 cells, and CXCR4 overexpression partially rescues the proliferative effects of miR-622 inhibition in SK-Hep1 and SNU448 cells (*n*=3). Student's *t*-test, *P<0.05. Error bars in panels are defined as s.d. (**c**) Transwell migration assay showing that CXCR4 inhibition partially reverse the migration effects of anti-miR-622 promotion in Huh7 and PLC/PRF/5 cells, and CXCR4 overexpression partially rescues the proliferative effects of miR-622 inhibition in SK-Hep1 and SNU448 cells (*n*=3). Student's *t*-test, *P<0.05. Error bars in panels are defined as s.d.

CXCR4 upregulation by anti-miR-622 was prevented using siRNAs before assessment of cell growth and migration. Hepatoma cells were transfected with CXCR4 or control siRNA before transfection with anti-miR-622 followed by assessment of cell growth and migration, respectively. CXCR4 knockdown with siRNA was confirmed by immunoblotting (Fig. 5a and Supplementary Fig. 4a,b). Inhibition of miR-622 significantly promoted growth and migration of Huh7 and PLC/PRF/5 cells, however, CXCR4 knockdown prevented the increased growth and migration induced by anti-miR-622 expression (Fig. 5b,c and Supplementary Fig. 4d). Similar rescue to the above was obtained in SK-Hep1 and SNU448 cells transfected with miR-622 (Fig. 5b,c and Supplementary Fig. 4c,d). The above data show that inhibitory effects of miR-622 are partially mediated by targeting CXCR4.

### Loss of miR-622 occurs in HCC with epigenetic abnormalities

**Figure 6:**
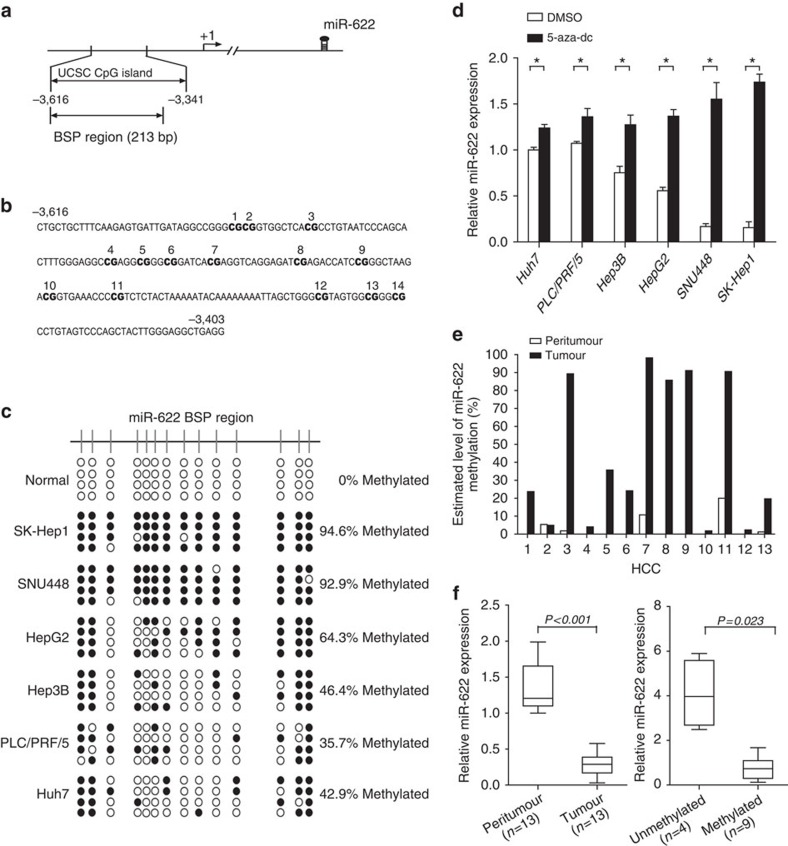
DNA methylation status of the miR-622 promoter. (**a**) Scheme depicting the genomic localization of miR-622. TSS is indicated with an arrow. Transcription start site (TSS) locations were predicted using miRStart (http://mirstart.mbc.nctu.edu.tw/)^44^. The regions analysed by BSP are indicated. (**b**) The sequence level detail of the miR-622 promoter regions (−3,616 to −3,403, 213 bp); the CpG dinucleotides within this region are numbered as 1–14. (**c**) The methylation status of the miR-622 promoter in the normal liver tissue (normal) and SK-Hep1, SNU448, HepG2, Hep3b, PLC/PRF/5 and Huh7 cell lines. The open and filled circles indicate the unmethylated and methylated CpGs, respectively. (**d**) Quantitative PCR (qPCR) analysis of miR-622 expression in indicated cells treated with 5 μmol 5-aza-dC for 72 h (*n*=3). Student's *t*-test, *P<0.05. Error bars in panels are defined as s.d. (**e**) The methylation status of the miR-622 promoter in 13 pairs of HCC and peritumour tissues. (**f**) qPCR analysis of miR-622 expression in 13 pairs of HCC and peritumour tissues (left) and in HCC tissues with or without methylation in miR-622 promoter (right; *n*=3). Student's *t*-test, *P<0.05. Error bars in panels are defined as s.d.

We next sought to investigate the molecular mechanism that mediates the downregulation of miR-622 in HCC. The miR-622 promoter is located in a typical CpG island, suggesting a possible involvement of DNA methylation in the regulation of miR-622 transcription (Fig. 6a,b). Although 100% of CpGs were unmethylated in normal liver, only 5.4–64.3% CpGs were unmethylated in six hepatoma cell lines: SK-Hep1, SNU448, HepG2, Hep3B, PLC/PRF/5 and Huh7 cells (Fig. 6c). The miR-622 expression was significantly increased from 2.5- to >11-fold when hepatoma cells with hypermethylated miR-622 promoter (SK-Hep1, SNU448 and HepG2) were treated with 5-aza-2′-deoxycytidine (5-aza-Dc) for 3 days (Fig. 6d). Furthermore, miR-622 methylation was assessed by bisulfite-sequencing PCR (BSP) in additional 13 pairs of HCC and peritumour tissues. Nine tumours showed more than 5% DNA methylation and only three peritumoural tissue was methylated (Fig. 6e). Peritumoural tissues had a higher expression of miR-622 as compared with HCC (Student's *t*-test *P*<0.001, Fig. 6f, left). Nine tumours were methylated and had lower expression levels of miR-622 in comparison with unmethylated tumours (Student's *t*-test *P*=0.023, Fig. 6f, right).

**Figure 7:**
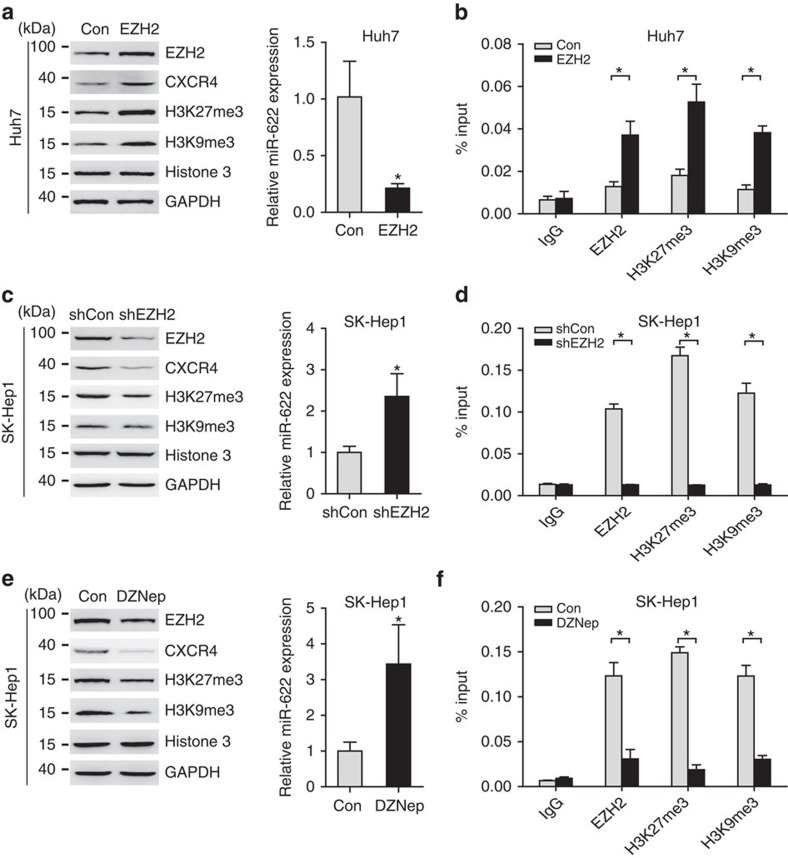
Expression of miR-622 is modulated with EZH2 in HCC cells. (**a**) Western blot analysis of EZH2, CXCR4, H3K27me3, H3K9me3, Histone 3 and GAPDH (left panel), quantitative PCR (qPCR) analysis of miR-622 (right panel) for Huh7 cells stably transfected with empty vector or EZH2 (*n*=3). Data are representative immunoblots of three independent assays. Student's *t*-test, *P<0.05. Error bars in panels are defined as s.d. (**b**) CHIP–qPCR analysis of EZH2, H3K27me3 and H3K9me3 enrichment at upstream of miR-622 for above-mentioned Huh7 cells (*n*=3). Student's *t*-test, *P<0.05. Error bars in panels are defined as s.d. (**c**) Western blot analysis of EZH2, CXCR4, H3K27me3, H3K9me3, Histone 3 and GAPDH (left panel), qPCR analysis of miR-622 (right panel) for SK-Hep1 cells stably transfected with nonspecific shRNA (shCon) or EZH2-specific shRNA (shEZH2; *n*=3). Data are representative immunoblots of three independent assays. Student's *t*-test, *P<0.05. Error bars in panels are defined as s.d. (**d**) CHIP–qPCR analysis of EZH2, H3K27me3 and H3K9me3 enrichment at upstream of miR-622 for above-mentioned SK-Hep1 cells (*n*=3). Student's *t*-test, *P<0.05. Error bars in panels are defined as s.d. (**e**) Western blot analysis of EZH2, CXCR4, H3K27me3, H3K9me3, Histone 3 and GAPDH (left panel), qPCR analysis of miR-622 (right panel) for SK-Hep1 cells without or with DZNep (10 μM) treatment for 72 h (*n*=3). Data are representative immunoblots of three independent assays. Student's *t*-test, *P<0.05. Error bars in panels are defined as s.d. (**f**) CHIP–qPCR analysis of EZH2, H3K27me3 and H3K9me3 enrichment at upstream of miR-622 for SK-Hep1 cells without or with DZNep treatment (*n*=3). Student's *t*-test, *P<0.05. Error bars in panels are defined as s.d.

Changes in DNA methylation could also be associated with the subsequent acquisition of other histone modifications. We further performed chromatin immunoprecipitation (CHIP) to evaluate repressive histone hallmarks, including trimethylated H3K9 (H3K9me3) and trimethylated H3K27 (H3K27me3). The results showed higher levels of methylation at H3K9 and H3K27 in a broad area upstream of the miR-622 coding region in SK-Hep1 cells (Supplementary Fig. 5a,b). These data allowed us to hypothesize that histone methylation, especially those of EZH2-dependent H3K27me3, may contribute to miR-622 repression. To confirm our hypothesis, we performed EZH2 overexpression and knockdown in hepatoma cells. As expected, overexpression of EZH2 led to an increase in CXCR4 expression and decrease in the levels of miR-622 in Huh7 cells (Fig. 7a). Furthermore, CHIP assays showed that EZH2 occupied in the upstream region of miR-622, which is concomitant with the increase in H3K27me3 and H3K9me3 levels (Fig. 7b). We confirmed an EZH2 knockdown using a specific shRNA (Supplementary Fig. 2c). Knockdown of EZH2 resulted in a great increase in the levels of miR-622 and decrease in the CXCR4 expression in SK-Hep1 cells (Fig. 7c). CHIP assay indicated that decreased EZH2, H3K27me3 and H3K9me3 occupancy in the upstream regions of miR-622 (Fig. 7d). Similarly, effects were observed during inhibition of EZH2 activity in SK-Hep1 cells by DZNep (Fig. 7e,f). These data indicate a link between epigenetic regulation and miR-622 transcription in hepatoma cell lines.

### EZH2 regulates CXCR4 by controlling miR-622 expression

**Figure 8:**
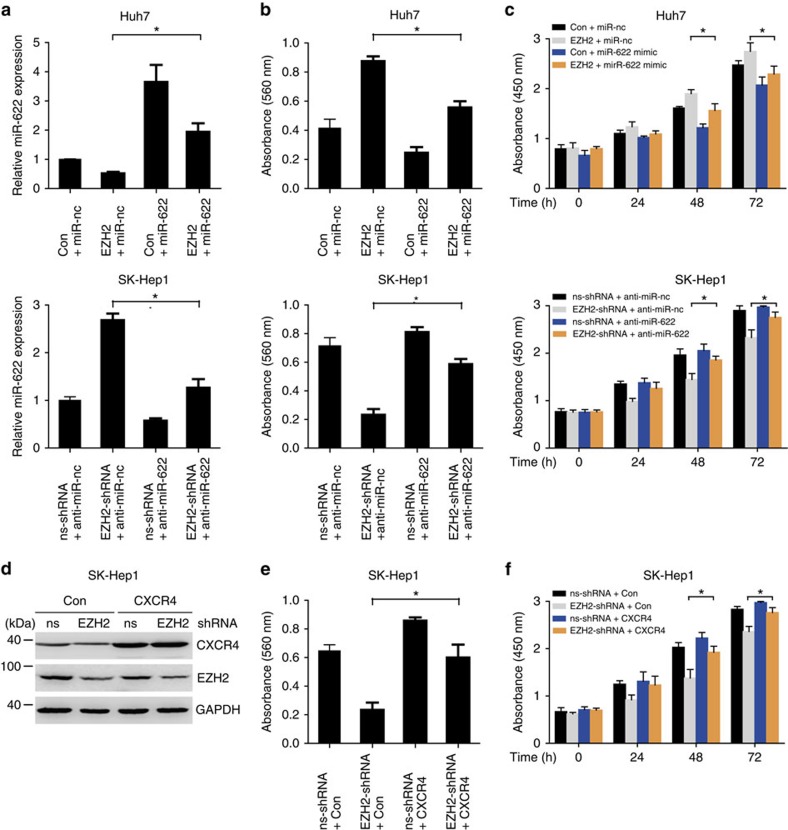
EZH2 regulates CXCR4 by controlling miR-622 expression. (**a**) Quantitative PCR analysis of miR-622 for Huh7 cells cotransfected with or without miR-622 and CXCR4, and SK-Hep1 cells cotransfected with or without EZH2-shRNA and anti-miR-622 inhibitor (*n*=3). Student's *t*-test, *P<0.05. Error bars in panels are defined as s.d. (**b**) Transwell migration assay showing that miR-622 partially reverse the migration effect of EZH2 promotion in Huh7 cells, and anti-miR-622 inhibitor partially rescues the migration effect of EZH2-shRNA inhibition in SK-Hep1 cells (*n*=3). Student's *t*-test, *P<0.05. Error bars in panels are defined as s.d. (**c**) Growth assay showing that miR-622 partially reverse the proliferative effects of EZH2 promotion in Huh7 cells, and anti-miR-622 inhibitor partially rescues the proliferative effects of EZH2-shRNA inhibition in SK-Hep1 cells (*n*=3). Student's *t*-test, *P<0.05. Error bars in panels are defined as s.d. (**d**) Western blot analysis of CXCR4, EZH2 and GAPDH in SK-Hep1 cells stably transfected with EZH2-shRNA cotransfected with or without CXCR4. Data are representative immunoblots of three independent assays. (**e**) Transwell migration assay showing that CXCR4 overexpression partially rescues the proliferative effects of EZH2-shRNA inhibition in SK-Hep1 cells (*n*=3). Student's *t*-test, *P<0.05. Error bars in panels are defined as s.d. (**f**) Growth assay showing that CXCR4 partially rescues the proliferative effects of EZH2-shRNA inhibition in SK-Hep1 cells (*n*=3). Student's *t*-test, *P<0.05. Error bars in panels are defined as s.d.

On the basis of our findings, we considered an aspect of the biological communication between epigenetic silencing and CXCR4 expression through miR-622 regulation. EZH2 knockdown in SK-Hep1 cells resulted in reduction of CXCR4 expression, which is consistent with those of miR-622 overexpression. Then, we tested whether exogenous manipulation of miR-622 could inhibit the effects of EZH2. We restored miR-622 expression in Huh7 cells, but inhibited miR-622 expression in SK-Hep1 cells (Fig. 8a). Indeed, enhancement of proliferation and migration induced by EZH2 overexpression was partially inhibited by treatment with miR-622 mimic in Huh7 cells. On the other hand, repression of proliferation and migration induced by knockdown of EZH2 was partially restored by treatment with miR-622 inhibitor in SK-Hep1 cells (Fig. 8b,c and Supplementary Fig. 6a,b). Additional expression of CXCR4 rescued the inhibition of proliferation and migration induced by EZH2 knockdown in SK-Hep1 cells (Fig. 8d–f and Supplementary Fig. 6c). These results suggest that EZH2-mediated miR-622 suppression leads to CXCR4 activation.

### Clinical correlation of CXCR4 with miR-622 EZH2

**Figure 9:**
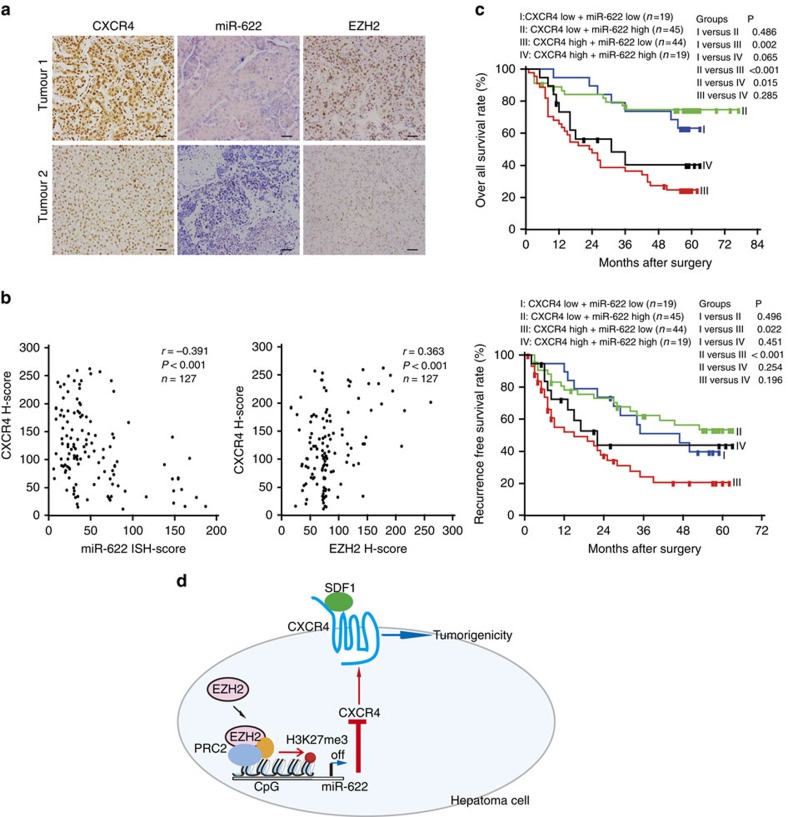
EZH2/miR-622/CXCR4 pathway correlates with poor prognosis in HCC patients. (**a**) Representative immunohistochemistry (IHC) staining with CXCR4, EZH2 and miRNA *in situ* hybridization (ISH) staining with miR-622 (scale bar, 50 μm). (**b**) Scatter plots showing the negative correlation between CXCR4 IHC score and miR-622 ISH score, and positive correlation between CXCR4 and EZH2 IHC score in HCC. Pearson's coefficient tests were performed to assess statistical significance. (**c**) Kaplan–Meier overall survival analysis curve (upper panel) or RFS analysis curve (lower panel) is shown for high- or low-risk survival group in 127 HCC patients. High CXCR4 expression and simultaneously low miR-622 level are significantly associated with both poorest overall survival and RFS. *P* value is determined by log-rank test. (**d**) Proposed model for CXCR4 upregulation in hepatoma cells. EZH2 are linked to CXCR4 activation via miR-622 regulation.

Given that miR-622 might regulate CXCR4 expression in HCC, miRNA *in situ* hybridization and immunohistochemical analysis were done to evaluate the relationship between miR-622 and CXCR4 expression in HCC (*n*=127). miR-622 level was inverse correlated with CXCR4 expression in HCC tissues (Pearson's coefficient test *r*=−0.391 (*P*<0.001), Fig. 9a,b). At the meanwhile, CXCR4 expression was positively correlated with EZH2 expression in HCC tissues (Pearson's coefficient test *r*=0.363 (*P*<0.001), Fig. 9a,b). Considering the inverse correlation between CXCR4 and miR-622 in the HCC tumours, we further evaluated their combined influence on patient outcome. Patients were classified into four groups, using their median as the cutoff. I, CXCR4 low and miR-622 low (*n*=19); II, CXCR4 low and miR-622 high (*n*=45); III, CXCR4 high and miR-622 low (*n*=44); and IV, CXCR4 high and miR-622 high (*n*=19). Patients in group III showed significantly worse overall survival and RFS than those in groups I and II (both log-rank test *P*<0.050, Fig. 9c). These data confirmed that EZH2/miR-622 pathway correlated with CXCR4 expression and was clinical relevant in HCC (Fig. 9d).

## Discussion

Inflammation drives different mechanisms involved in tumorigenesis and progression, including proliferation of tumour cells, angiogenesis and metastasis^18^. These mechanisms are, in part, driven by secreted molecules such as CXCL12, which plays multiple roles in tumour pathogenesis^19^. Although they were first described to be produced by bone marrow stromal cells, they are also secreted by tumour cells of different origin, including hepatocellular carcinoma cells^19^. The CXCR4/CXCL12 has multiple roles in the pathogenesis of HCC, and can modulate cell growth, migration and survival via both autocrine and/or paracrine mechanisms^5^. A number of studies have demonstrated correlations between high CXCR4 expression and aggressive tumour behaviour and poor prognosis^6^,^8^,^20^. Therapeutic intervention with CXCR4 signal activation could be used as a promising strategy against hepatocellular carcinoma after curative resection. Administration of CXCR4 antagonist has been found to inhibit tumour growth and metastasis^4^,^21^,^22^,^23^. Discrepancies between our results and the report by Duda *et al*., which suggest that AMD3100 was ineffective in HCC tumorigenesis, probably rely on that the CXCR4 antagonist intervention on endogenous CXCR4 high-expression HCC cell line, SK-Hep1, in tumorigenesis *in vivo* in the present study^24^. There are multiple classes of CXCR4 antagonists in clinical trials^4^,^25^. These include: small-molecule inhibitors (for example, AMD3100), small modified peptides (for example, BTK140), antibodies (for example, ALX-0651) and modified CXCL12 antagonists (for example, CTCE-9908). Given the accumulating evidence for the critical role of CXCR4 in cancer, such compounds are currently being tested in early-phase clinical trials^4^,^25^. Our studies suggest that HCC patients should be included in these trials.

In HCC patients, CXCR4 was detected in HCC tissues, but not in normal hepatic tissues. CXCR4 expression significantly correlated with progressed local tumours (T-status), lymphatic metastasis and distant dissemination, as well as with a decreased survival^6^,^7^,^26^,^27^. Nonetheless, conflicting data were reported that the CXCL12–CXCR4 are detected in sinusoidal endothelial cells in HCC tissue, and their expression are significantly higher than in non-HCC tissues^20^. Furthermore, Zhou reported that CXCR4 nuclear localization can be used to identify patients with HCC at high risk for developing lymph node metastasis^8^. The discrepancy may lie in the heterogeneity of HCC and different detection methods applied.

The mechanism underlying CXCR4 overexpression in HCC is unclear at present. A number of studies have demonstrated upregulation of CXCR4 in HCC tissues, while CXCR4 mRNA expression reduced or remain no differences^6^,^28^. This means that CXCR4 may be regulated by post-transcriptional level in HCC. We provide definitive evidence for the notion qthat miR-622 negatively regulates CXCR4 expression. Here miR-622 is verified to be frequently decreased in HCC tissues, and inversely correlated with the survival of HCC patients, outlining a potential marker for predicting the prognosis of HCC patients. Furthermore, the therapeutic role of miR-622 in HCC remains to be elucidated. In the meanwhile, we cannot exclude other potential miRNA participating in CXCR4 regulation.

The miRNA regulation involves multiple steps, including miRNA maturation, genetic deletion and epigenetic deregulation^29^,^30^. In particular, polycomb group proteins have central functions in cellular development and regeneration by controlling histone methylation, especially at histone H3 Lys27 (H3K27), which induces chromatin compaction^31^. Alterations of PcG genes directly modulate the trimethylation of H3K27 and thus affect the epigenome of HCC, which is crucial for controlling the HCC cell phenotype^32^. Oncogene polycomb group protein EZH2 in HCC is highly correlated with tumour progression^33^. We show that EZH2 epigenetically represses miR-622 expression by facilitating H3K27me3 trimethylation. Consistently, miR-101 was epigenetically silenced by EZH2 overexpression in HCC^34^.

Recent studies have suggested unique expression profiles of miRNAs in HCC^17^,^35^, but loss of miR-622 has not been focused. Besides the variations of technological methodologies and sample origin, one main reason for this inconsistency is possibly that the liver is composed by a heterogeneous population of parenchymal cells, kupffer cells, stellate cells, bile duct cells, fibroblasts and inflammatory cells^36^. HCC samples of patients with different aetiologies usually with different cell activities and proportions may result in artifact of miRNA profiles. In the present study, we also discovered many dysregulated miRNAs in HCC that were consistently reported before (such as miR-139-5p and miR-1) (refs 15, 16).

In conclusion, the coordinated expression of EZH2/miR-622/CXCR4 may be predictive of worse prognostic in patients with HCC. Our findings also highlight the therapeutic potential of CXCR4 in HCC treatment, and support the development of effective therapeutic strategies that target CXCR4 by a pharmacological approach. Therefore, CXCR4 could be a therapeutic target and a valuable prognostic marker for HCC.

## Methods

### Cell culture and generation of stable cell lines

Human hepatoma cell lines Huh7, PLC/PRF/5, Hep3B, HepG2, SNU448 and SK-Hep1 were obtained directly from Shanghai Cell Bank of Chinese Academy of Sciences (Shanghai, China), where they were authenticated by short tandem repeat profiling, and characterized by mycoplasma detection and cell vitality detection. Cell lines were cultured in Dulbcco's Modified Eagle's Medium (DMEM) supplemented with 10% fetal bovine serum at 37 °C in a humidified 5% CO_2_ incubator. All cell lines were placed under cryostage after they were obtained from the bank and used within 6 months of thawing fresh vials. To generate cell populations stably expressing CXCR4, EZH2 or control plasmids were transfected into Huh7 cells with FuGENE HD Transfection Reagent (Roche Applied Science, Mannheim, Germany) according to the manufacture's protocol. After 24 h of transfection, stable transfectants were selected in medium containing 800 μg ml^−1^ G418 (Sigma-Aldrich, St Louis, MO) for 4 weeks. Control-shRNA, CXCR4-shRNA or EZH2-shRNA-engineered SK-Hep1 cells were generated by transducing lentiviral particles containing pRS-CXCR4 human short hairpin RNAs (shRNA; TR313630A, TR313630B and TR313630C; OriGene, Rockville, MD; Supplementary Table 4), pRS-EZH2 human shRNAs (TR304713A, TR304713B and TR304713C, Supplementary Table 4) or pRS-non-silencing Luc-shRNA, and selected with puromycin 2 μg ml^−1^ for 2 weeks. Pooled populations of transduced cells were used to avoid selection for clonal variants.

### Human HCC tissues

Paired (*n*=127) HCC and adjacent non-tumour tissues were collected from Nantong Tumor Hospital (Jiangsu, China) from January 2003 to February 2005 after approval by the Research Medical Ethics Committee of Fudan University. Informed consent was provided by each patient. All cases were in accord with the following criteria: diagnosed by postoperative histopathology; complete follow-up data available; no extrahepatic metastasis; no other malignant disease; and no preoperative anticancer therapy. Curative hepatectomy was defined as complete resection of all tumour nodules and the cut surface being free of cancer by histology examination, having no cancerous thrombus in the portal vein and having no extrahepatic metastasis^37^. Patients were redesigned according to the 7th edition TNM classification system of the American Joint Committee on Cancer/International Union Against Cancer. Overall survival was calculated from the data of surgery to the data of death or the last follow-up. Recurrence was defined as the emergence of one or several liver enhancing foci at computed tomography or magnetic resonance imaging^38^. None of these patients died from operative complications or other factors. The mean age of the patients was 51 years (range, 17–79 years). The last follow-up was May 2012, with a median follow-up of 34 months (range, 1–82 months).

### Plasmid construction and transfection

Expression plasmid encoding wild-type CXCR4 was kindly provided by Dr Ann Richmond (Vanderbilt University, Nashiville, TN). Expression plasmid encoding wild-type EZH2 was generated as previous described^39^. The 3′-UTR of CXCR4 was amplified from HepG2 and cloned into the downstream region of a luciferase gene in a modified pGL3 control vector (Promega, Madison, WI). The potential miR-622 binding site in the 3′-UTR of CXCR4 was mutated by the overlap extension PCR method. The primer sets are listed in Supplementary Table 5. The DNA constructs were verified by sequencing. Transient and stable transfections with various plasmids were performed as manuscript provided. The miRNA mimic and anti-miRNA molecule of miR-302c, miR-139-5p, miR-9, miR-206, miR-622, or nonspecific control miRNA (miR-nc mimic or anti-miR-nc; Invitrogen, Carlsbad, CA) 100 nM was transfected into hepatoma cells.

### Cell proliferation and colony formation assay

Cell proliferation was determined by the Cell Counting Kit-8 (Dojindo, Kamimashiki-gun Kumamoto, Japan) according to the manufacturer's instructions. Cell proliferation rate was assessed by measuring the absorbance at 450 nm with the Universal Microplate Reader (BioTEK Instruments, Minneapolis, MN). Anchorage-independent growth ability was measured by using soft agar colony formation assay. A total of 10 × 10^3^ cells were resuspended in DMEM containing 0.3% noble agarose (Promega Corporation, Madison, WI). This suspension was laid over DMEM containing 0.6% noble agarose in six-well plates and further overlaid with DMEM. The plates were then incubated for 14 days in a 5% CO_2_ incubator at 37 °C, with replenishment of medium every other day. Colonies were imaged using Nikon ECLIPSE TE300 and macroscopically visible colonies in three randomly chosen fields per well were counted for quantification.

### Cell migration assay

A QCM 24-well colorimetric cell migration assay (Chemicon, Temecula, CA, USA) was used for this experiment. Hepatoma cell lines were trypsinized and seeded at a concentration of 1 × 10^6^ cells per ml in serum-free DMEM. The 24-well plates were then incubated for 24 h in a humidified 5% enriched CO_2_ atmosphere. Cells that migrated through the 8-μm pore membranes, located at the bottom of every well insert, were stained and eluted. All experiments were performed in three replicates. Cell migration was assessed by measuring the absorbance at 560 nm with the Universal Microplate Reader (BioTEK Instruments, Minneapolis, MN).

### *In silico* prediction for miR targeting of CXCR4 3′-UTR

Three algorithms were used to predict potential miR targeting of 3′-UTR of CXCR4: Pictar (http://pictar.mdc-berlin.de/); Targetscan (http://www.targetscan.org/); and MiRanda (http://www.microrna.org/microrna/home.do)^40^. To reduce the number of false positives, only the miRs that were predicted by at three algorithms were subsequently validated.

### Luciferase activity assay

Luciferase activity was assayed with Dual-Luciferase Reporter Assay System (Promega, Madison, WI) according to the manufacturer's instructions. The co-expressed Renilla luciferase activity was used for the normalization of transfection efficiency.

### Real-time PCR with reverse transcription

Total RNA from frozen tissue specimens and cultured cells was extracted using TRIzol reagent (Invitrogen, Carlsbad, CA) according to the manufacturer's instructions. RNA quantity and quality were determined by a NanoDrop ND-1000 Spectrophotometer (NanoDrop Technologies, Wilmington DE). Furthermore, mature miRNA levels were measured by using an NCode miRNA First-strand cDNA synthesis and real-time PCR Kit (Invitrogen, Carlsbad, CA). U6 snRNA (RNU6B) served as an endogenous control. Expression of the primary miR-622 transcript was analysed using a TaqMan Pri-miRNA assay (Assay ID Hs03304667_pri; Applied Biosystems). Primer sets are listed in Supplementary Table 5. Real-time PCR was performed on the Applied Biosystems 7300 Real-Time PCR system using SYBR Green dye (Applied Biosystems, Foster City, CA) as described by the manufacture. All determinations were performed in triplicate and in at least three independent experiments. The 2^−ΔΔ^Ct method was applied to estimate relative transcript levels.

### Western blot

Specimen tissues or tumour cells were homogenized in modified lysis buffer (50 mM Tris-HCl (pH7.5), 100 mM NaCl, 50 mM NaF, 1 mM Na_3_VO_4_, 30 mM sodium pyrophosphate, 0.5% NP-40 and 0.5 mM phenylmethyl sulfonyl fluoride (Sigma-Aldrich) supplemented with EDTA-free protease inhibitor cocktail (Roche). Lysis were resolved by SDS–PAGE and transferred to a PVDF membrane (Millipore, Billerica, MA). The membranes were blocked with 5% non-fat dry milk, incubated with primary antibodies (Supplementary Table 6) for overnight at 4 °C and subsequently reacted with horseradish peroxidase-conjugated secondary antibodies (sc-2004 and sc-2005, Santa Cruz Biotechnology) for 1 h at room temperature. Bands were visualized using the ECL detection system (GE Healthcare, Chalfont St Giles, UK). Uncropped images of all blots are in Supplementary Fig. 7.

### DNA methylation analysis

DNA was isolated using the proteinase K/phenol extraction method. Bisulfite conversion was carried out using 1 μg of DNA using an Epitect Bisulfite Kit (Qiagen). Bisulfite-treated DNA was amplified with BSP primers located in the miR-622 promoter, forward: 5′- GAAATTGTTGTTTTTAAGAGTGATTGATA -3′ and reverse: 5′- CAACCTCCCAAATAACTAAAACTACA -3′. PCR products were cloned using the pGEM-T Easy Vector system (Promega, Madison, WI). Four individual clones were sequenced. The region assessed by BSP included 14 CpG sites from the miR-622 promoter and average methylation from individual clones was calculated as a percentage of the number of methylated CpG sites over the number of total CpG sites sequenced.

### Chromatin immunoprecipitation assay

CHIP assay was performed using the EZ-CHIP chromatin immunoprecipitation kit (Millipore, Billerica, MA). following the manufacturer's protocol. Immunoprecipitate (IP) complexes were immunoprecipitated with an anti-EZH2, H3K27me3, H3K9me3 antibodies or rabbit IgG antibody (Supplementary Table 6) overnight at 4 °C. The captured genomic DNA was obtained and used for quantitative PCR analysis. Ten per cent of total genomic DNA from the nuclear extract was used as input. The primers used for detection of miR-622 promoter sequence as follows: P1 forward: 5′- GAAGCATCTCCAGACCGAGA -3′, reverse: 5′- CGAGTGGCCGACTCTGGA -3′; P2 forward: 5′- GATTACAGGCATGCGCCAC -3′, reverse: 5′- CACCATAGCTGGACACCTGG -3′; P3 forward: 5′- CGCTGGCTCATGCCTGTAAT -3′, reverse: 5′- CACCACCATGCCTGGCTAAT -3′; P4 forward: 5′- GGAAGCATCTCCAGACCGAG -3′, reverse:5′- CGAGTGGCCGACTCTGG -3′. Amplification efficiency was calculated, and the data were expressed as enrichment related to input.

### *In situ* hybridization

Paraffin-embedded sections were deparaffinized and rehydrated by an ethanol series. Slides were quenched endogenous peroxidase activity with 3% H_2_O_2_ for 30 min. Following digestion by proteinase K for 5 min, slides were fixed in 4% paraformaldehyde and rinsed in PBS. Slides were incubated in hybridization buffer at 60 °C for 2 h, and incubated with miR-622 or scrambled miRNA control probes (50 nM; digoxigenin-labelled LNA probes, Exiqon, Vedbaek, Denmark) at 60 °C overnight. Stringent wash buffer (50% formamide in 2 × SSC and PBS plus Tween-20 (PBST)) was used. Alkaline phosphatease substrate (nitroblue tetrazolium/5-bromo-4-chloro-3-indolyl- phosphate was used for the alkaline phosphate reaction, and the slides were mounted in aqueous mounting medium. Scoring was measured according to the following criterion: 0=absent cell cytoplasm staining; 1=weak cell cytoplasm staining; 2=moderate cell cytoplasm staining; 3=strong cell cytoplasm staining, and the ISH score of percentage multiplying the intensity was recorded^41^. The median value of the ISH score was chosen as the cutoff criterion to dichotomize into high- and low-expression subgroups, and the median value of miR-622 ISH score is 36.

### Tumour xenograft experiments

Four- to five-week-old male athymic nude (*Foxn1*^nu/nu^, BALB/c background) mice were purchased from Shanghai Laboratory Animal Center (Chinese Academy of Sciences, Shanghai, China). Mice age 5–6 weeks were injected subcutaneously in the flank on each side with 1 × 10^7^ viable SK-Hep1, SK-Hep1-shCon, SK-Hep1-shCXCR4, Huh7-Con or Huh7-CXCR4 cells. Ten mice per cell line were used. Tumour size was monitored by digital caliper. Tumour volume=(*L* × *W*^2^)/2, where *L* is length at the widest point of the tumour and *W* is the maximum width perpendicular to *L*. When the tumours reached ∼80 mm^3^ in diameter, the mice were randomized into treatment groups. The mice were administered subcutaneously daily with a solution AMD3100 (10 mg kg^−1^) or PBS for 3 weeks. Anti-CXCR4 neutralizing antibody (clones 12G5, BD Pharmingen, SanDiego, CA) or irrelevant antibody (IgG2a) (R&D System, Minneapolis, MN) was injected intraperitoneally (1 mg per injection) twice a week for 3 weeks. Mice were sacrificed if the volume of their tumour reached 2,000 mm^3^. All animal procedures were approved and performed in accordance with the guidelines of the Fudan University Animal Care and Use Committee (Permit No. 13022708).

### Tissue microarray and immunohistochemical staining

Haematoxylin and eosin staining was used to define the diagnostic area, and one representative core (6 mm) was obtained from each case using a tissue arrayer (Beecher Instruments, Silver Spring, MD). Tissue microarray sections (4 μm) was immunostained with antibodies to CXCR4 (dilution: 1:200, Supplementary Table 6), EZH2 (dilution: 1:400, Supplementary Table 6). Immunohistochemical evaluation was performed independently by two researchers (Y.L. and W.L.) blinded to the clinical data, and cases with discrepant grades were re-evaluated by discussion until consensus was achieved. We classified the IHC staining results into two categories according to subcellular localization of CXCR4, for example, cytoplasmic and nuclear. A semi-quantitative H-score ranged from 0 to 300 was calculated for each specimen by multiplying the distribution areas (0–100%) at each staining intensity level by the intensities (0: negative, 1: weak staining, 2: moderate staining and 3: strong staining)^42^. The median value of the H-score was chosen as the cutoff criterion to dichotomize into high and low expression subgroup. As a result, CXCR4 median H-score is 110 and EZH2 median H-score is 75.

### Statistical analysis

All quantified data represent a mean of triplicate samples±s.d.^43^ through analysing with GraphPad Prism 5 (GraphPad Software, La Jolla, CA) and assessing comparisons between different groups by the Student's *t*-test and one-way analysis of variance. The correlation between CXCR4 and clinicopathologic features was assessed using *χ*^2^ or Fisher's exact test with Stata software, version 12 (StataCorp, College Station, TX). Survival was calculated starting from the data of death or last follow-up. Survival curves were estimated using Kaplan–Meier method and log-rank test was used to compute differences between the curves. The correlation between CXCR4 with miR-622 and EZH2 staining by ISH and IHC, were determined using Pearson's coefficient test. Differences were considered significant at values of *P*<0.05. All statistical tests were two sided.

## Additional information

**How to cite this article:** Liu, H. *et al*. EZH2-mediated loss of miR-622 determines CXCR4 activation in hepatocellular carcinoma. *Nat. Commun.* 6:8494 doi: 10.1038/ncomms9494 (2015).

## Supplementary Material

Supplementary Information Supplementary Figures 1-7 and Supplementary Tables 1-6

## Figures and Tables

**Table 1:** Multivariate cox regression analyses of overall survival and RFS after surgery in 127 HCCs.

**Characteristics**	**OS**		**RFS**	
	**HR (95% CI)**	***P***	**HR (95% CI)**	***P***
Child-Pugh ( B versus A)	1.031 (0.415–2.559)	0.948	NA	NA
AFP, ng ml^−1^ (>20 versus ≤20)	1.280 (0.664–2.466)	0.464	NA	NA
Tumour encapsulation (none versus complete)	1.112 (0.629–1.963)	0.717	1.291 (0.758–2.201)	0.35
Tumour differentiation (III–IV versus I–II)	2.207 (1.244–3.918)	**0.007**	NA	NA
Tumour size, cm (>5 versus ≤5)	0.544 (0.208–1.419)	0.216	0.795 (0.312–2.026)	0.633
Vascular invasion (present versus absent)	2.104 (1.118–3.958)	**0.022**	1.542 (0.913–2.606)	0.107
BCLC (B+C versus 0+A)	4.652 (1.759–12.30)	**0.002**	2.119 (0.852–5.271)	0.108
TNM (III versus I+II)	2.603 (1.398–4.846)	**0.003**	1.293 (0.738–2.265)	0.372
CXCR4 (high versus low)	1.889 (1.040–3.431)	**0.038**	1.695 (1.008–2.853)	**0.048**

AFP, alpha-fetoprotein; ALT, alanine aminotransferase; BCLC, Barcelona Clinic Liver Cancer staging; CI, confidence interval; HBsAg, hepatitis B surface antigen; HR, hazard ratio; NA, not applicable; OS, overall survival.

Bold values indicate *P*<0.05, *P* values from cox regression analysis.
