# Author Correction: EZH2-mediated loss of miR-622 determines CXCR4 activation in hepatocellular carcinoma

**DOI:** 10.1038/s41467-021-26555-7

**Published:** 2021-11-05

**Authors:** Haiou Liu, Yidong Liu, Weisi Liu, Weijuan Zhang, Jiejie Xu

**Affiliations:** 1 Shanghai Key Laboratory of Female Reproductive Endocrine Related Diseases, Hospital of Obstetrics and Gynecology, Fudan University, Shanghai, 200011 China; 2 Department of Biochemistry and Molecular Biology, School of Basic Medical Sciences, Fudan University, Shanghai, 200032 China; 3 Department of Immunology, School of Basic Medical Sciences, Fudan University, Shanghai, 200032 China

Correction to: *Nature Communications* 10.1038/ncomms9494, published online 25 September 2015.

This Article contains an error in Figure 2. In Fig. 2c the AMD3100 image was inadvertently duplicated from the anti-CXCR4-Ab image. The incorrect images are shown below.
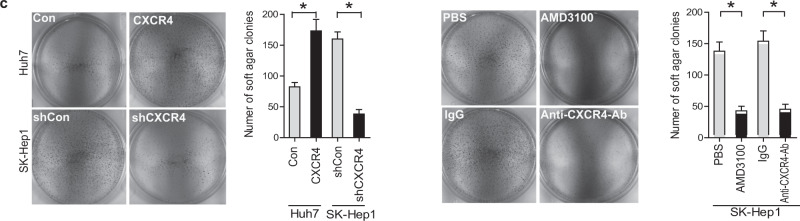


The correct images are shown below.
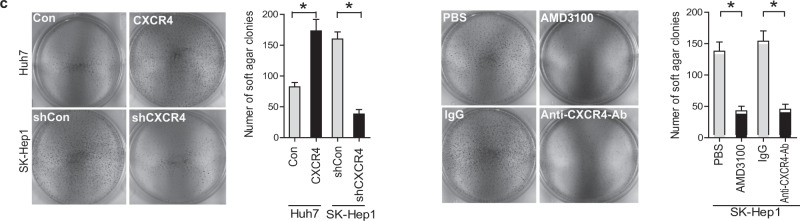


This error has not been corrected in the PDF or HTML versions of the Article.

