# Supplementary material for: EZH2-mediated loss of miR-622 determines CXCR4 activation in hepatocellular carcinoma
Source: Nat Commun. 2015 Sep 25;6:8494. doi: 10.1038/ncomms9494 (PMC4598861; doi:10.1038/ncomms9494)
Supplement: Supplementary Information — Supplementary Figures 1-7 and Supplementary Tables 1-6 [file ncomms9494-s1.pdf]

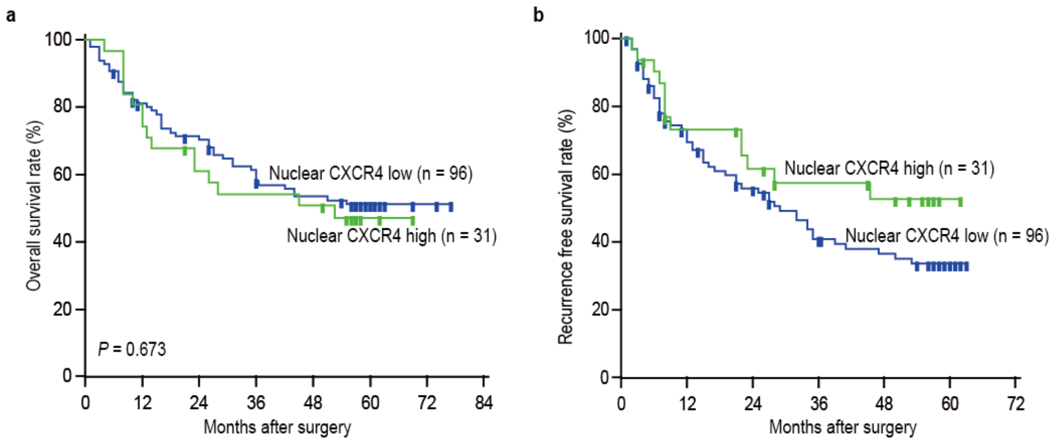

Supplementary Figure 1: Nuclear CXCR4 is not correlated with clinical outcome of HCC patients Kaplan-Meier plots indicate the overall survival (a) and recurrence free survival (b) for HCC patients categorized by nuclear CXCR4 expression (n = 31 for high nuclear CXCR4 group versus n = 96 low CXCR4 group), *P* value is determined by log-rank test. CXCR4 high , IHC score ≥ 110.

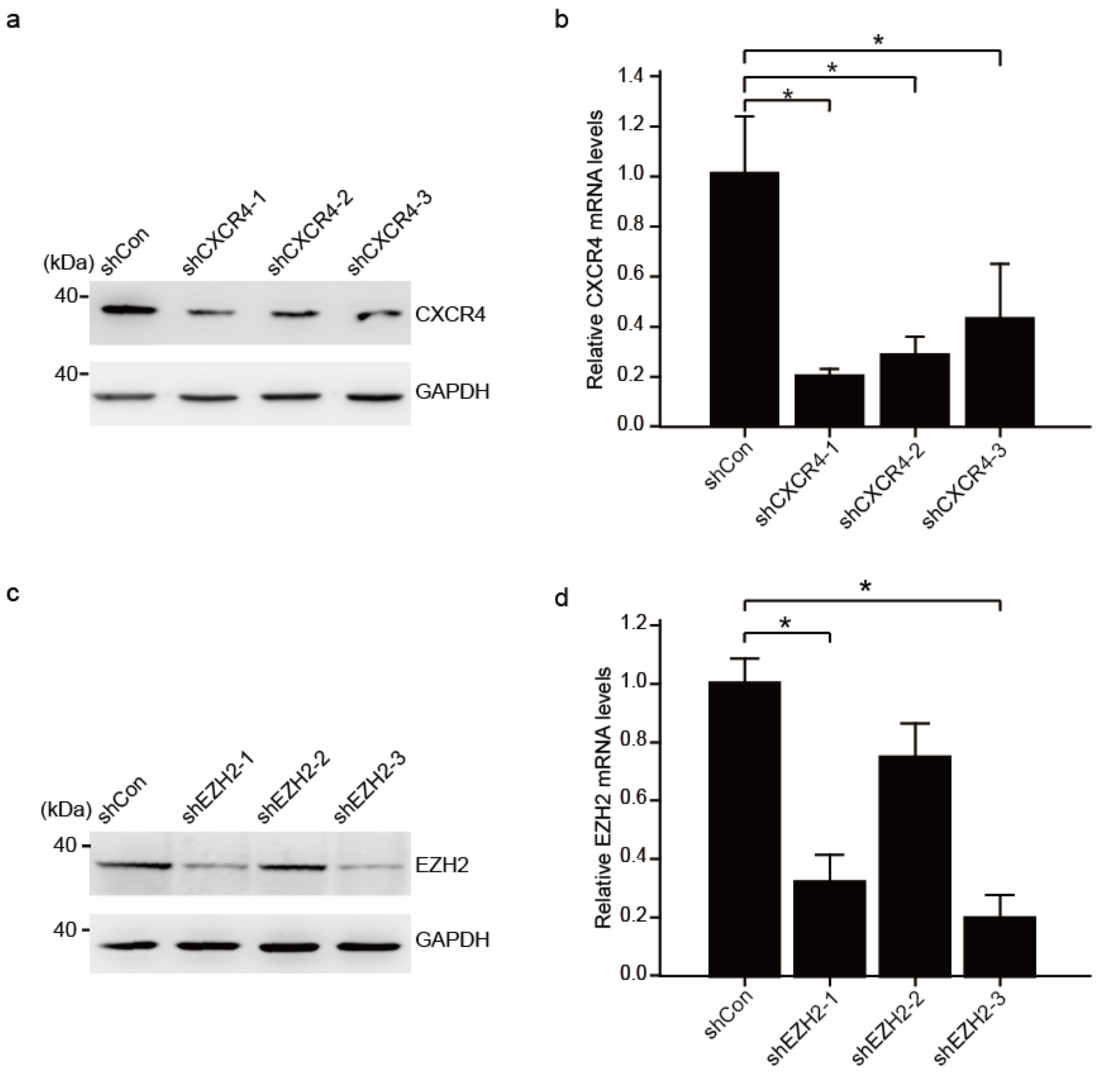

Supplementary Figure 2: shRNA-mediated knockdown of CXCR4 and EZH2 expression in SK-Hep1 cells (a) Western blot analysis of CXCR4 shRNA interference in SK-Hep1 cells. Data are representative immunoblots of three independent assays. **(b)** Real-time analysis of relative CXCR4 mRNA levels in above mentioned SK-Hep1 cells. (n=3 per group). Student's t-test * $P < 0.05$. Error bars in panels are defined as s.d. **(c)** Western blot analysis of EZH2 shRNA interference in SK-Hep1 cells. Data are representative immunoblots of three independent assays. **(d)** Real-time analysis of relative EZH2 mRNA levels in above mentioned SK-Hep1 cells. (n=3 per group). Student's t-test * $P < 0.05$. Error bars in panels are defined as s.d.

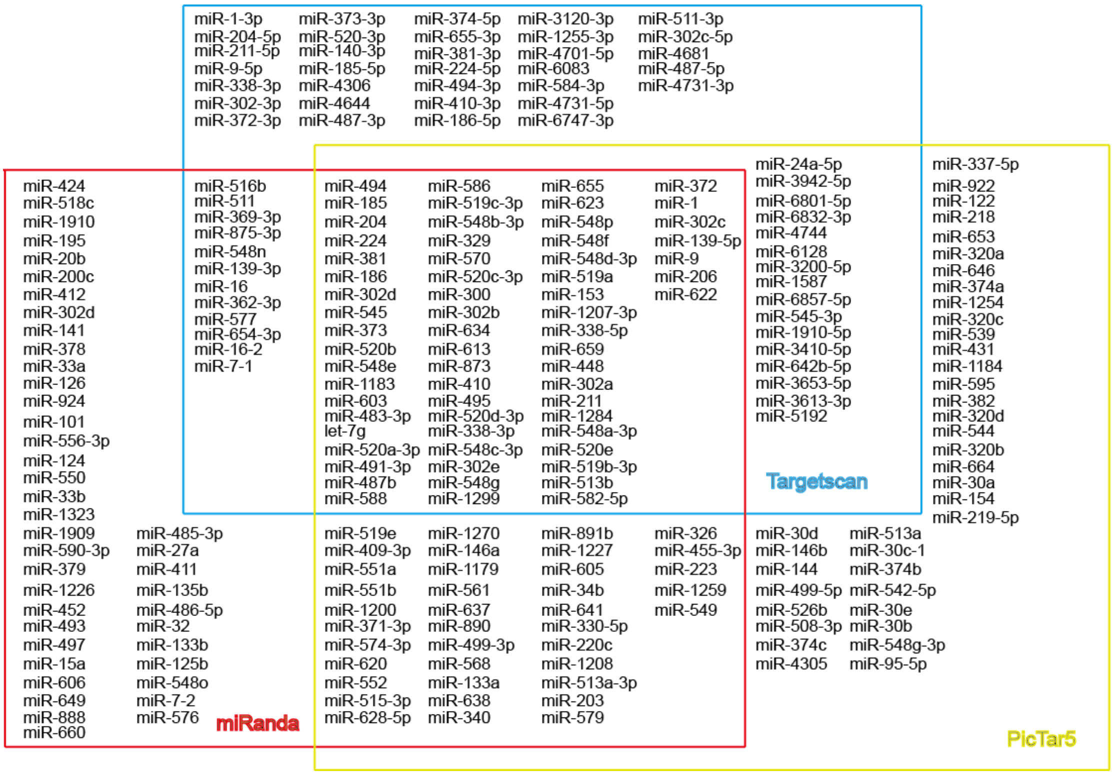

Supplementary Figure 3: Prediction of miRNAs target CXCR4. miRNAs target CXCR4 predicted by Targetscan, miRanda and Pictar5. miRNAs that were predicted by two or more algorithms are indicated.

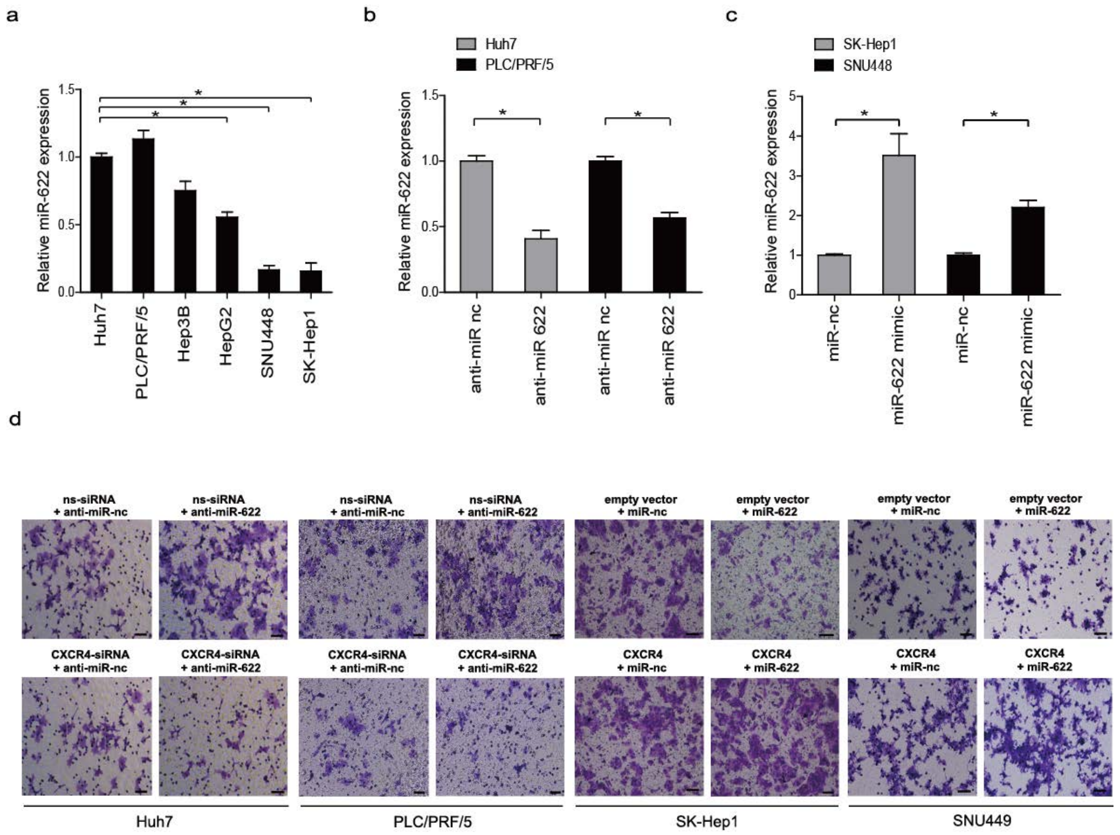

Supplementary Figure 4: related to Figure 5. CXC4 mediate the effects of miR-622 on hepatoma cell growth and migration. (a) Real-time analysis of relative miR-622 levels in hepatoma cell lines (*n*=3 per group). Student's *t*-test * $P < 0.05$. Error bars in panels are defined as s.d. (b) Real-time analysis of relative miR-622 levels in Huh7 and PLC/PRF/5 cells transfected with anti-miR-nc or anti-miR-622 (*n*=3 per group). Student's *t*-test * $P < 0.05$. Error bars in panels are defined as s.d. (c) Real-time analysis of relative miR-622 levels in SK-Hep1 and SNU448 cells transfected with miR-nc or miR-622 mimic (*n*=3 per group). Student's *t*-test * $P < 0.05$. Error bars in panels are defined as s.d. (d) Representative images depicting cell migration assay performed with 1×10^6 above mentioned hepatoma cells. Scale bar, 50μm.

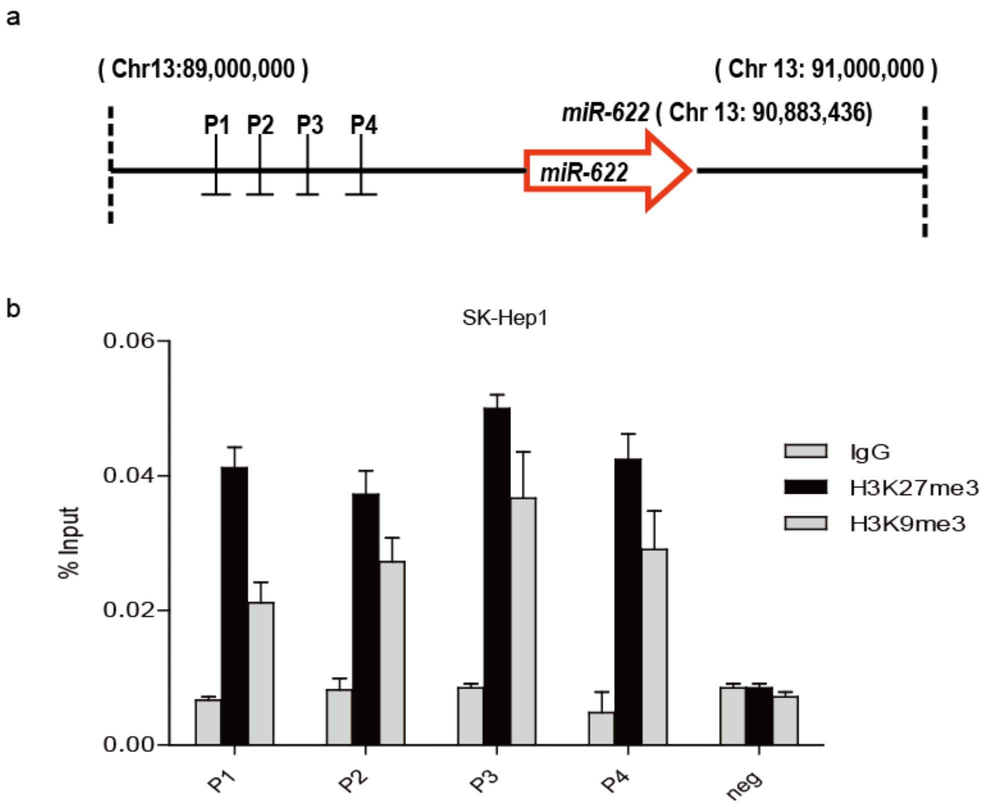

Supplementary Figure 5: Genomic location and H3K27me3, H3K9me3 binding at upstream of miR-622 (a) Schematic showing genomic location of miR-622. The upstream regions used for CHIP-PCR are indicated as short black lines. **(b)** CHIP-PCR showing percent (%) input of H3K27me3 at indicated locations upstream of miR-622 in SK-Hep1 cells. IgG was used as control. (*n* = 3 per group). Error bars in panels are defined as s.d.

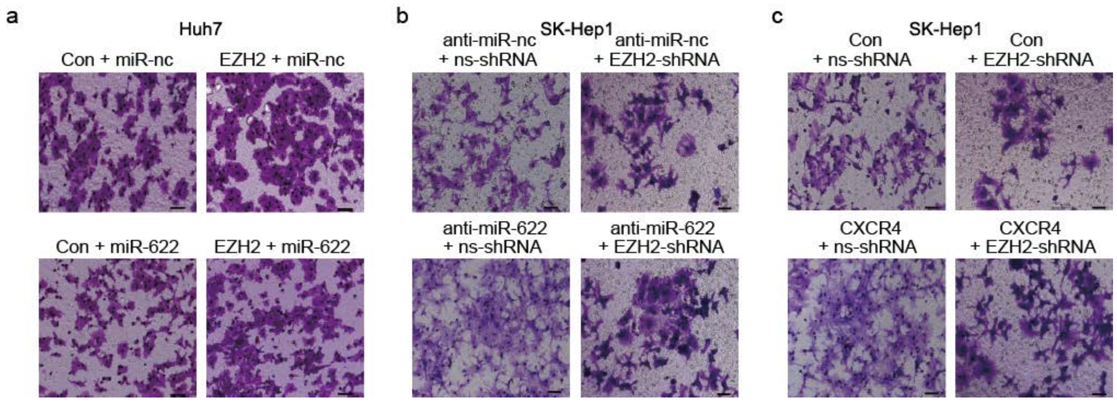

Supplementary Figure 6: related to Figure 8. EZH2 regulates CXCR4 by controlling miR-622 expression. (a) Representative images depicting cell migration assay performed with 1 × 10^6 Huh7 cells contransfected with or without miR-622 and CXCR4. (b, c) Representative images depicting cell migration assay performed with 1 × 10^6 SK-Hep1 Cells cotransfected with or without EZH2-shRNA and anti-miR-622 or CXCR4. Scale bar, 50μm.

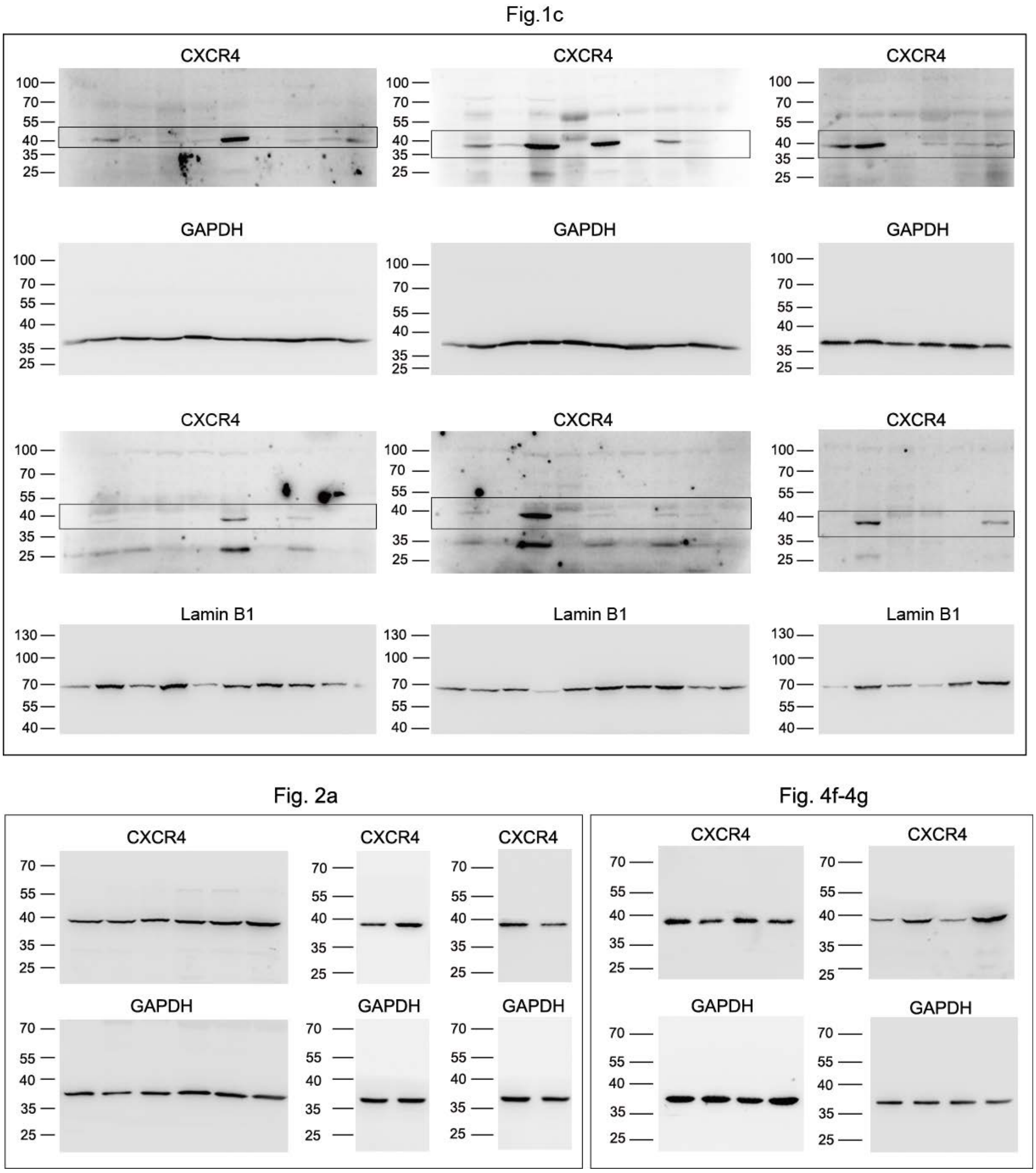

Supplementary Figure 7: Uncropped images of immunoblots.

Fig. 4b

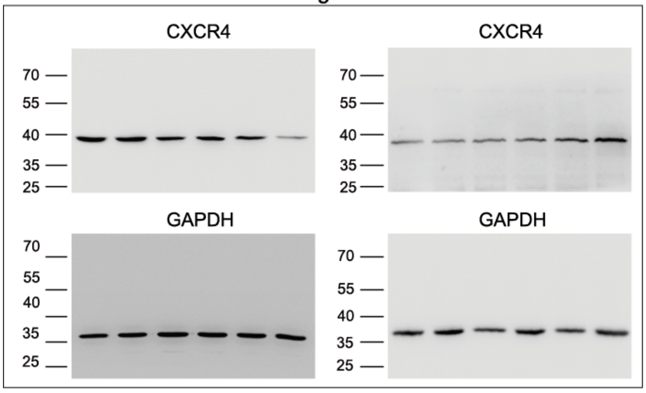

Fig. 5a

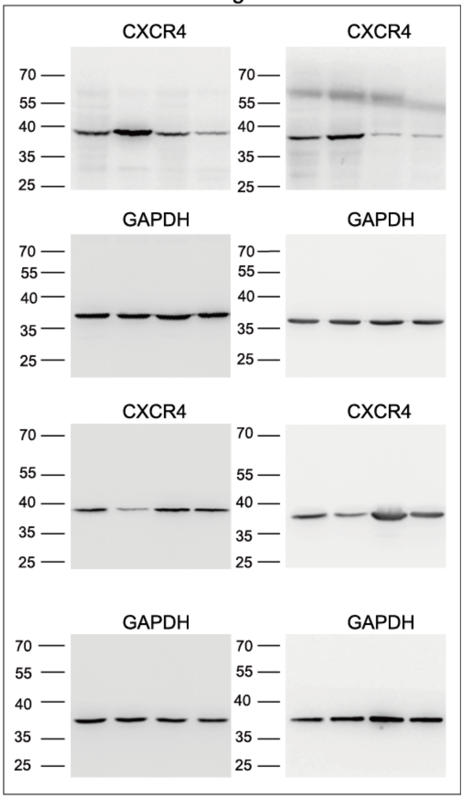

Fig. 7a

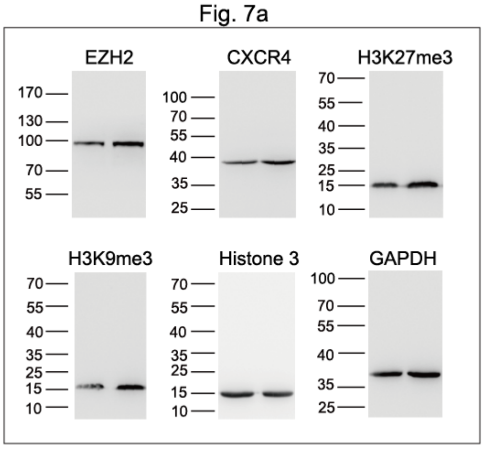

Fig. 7c

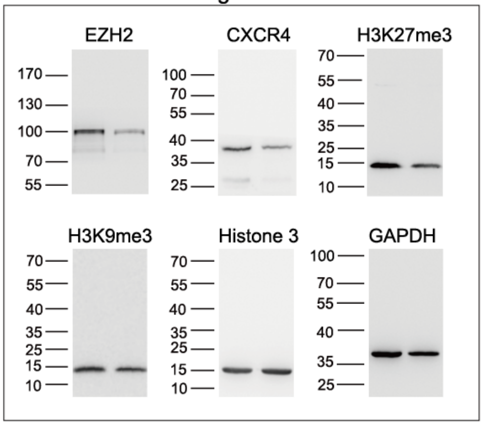

Fig. 7e

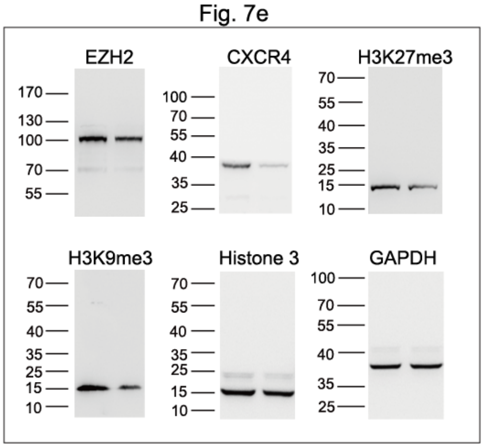

Supplementary Figure 7: Uncropped images of immunoblots (continued).

Fig. 8d

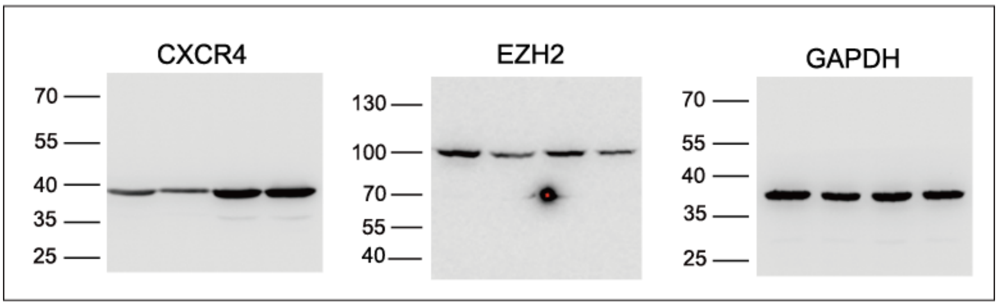

Supplementary Fig. 2a

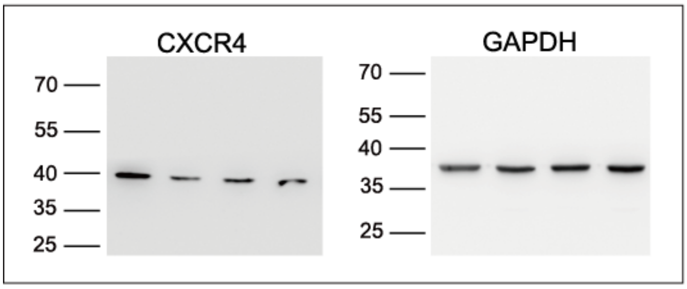

Supplementary Fig. 2c

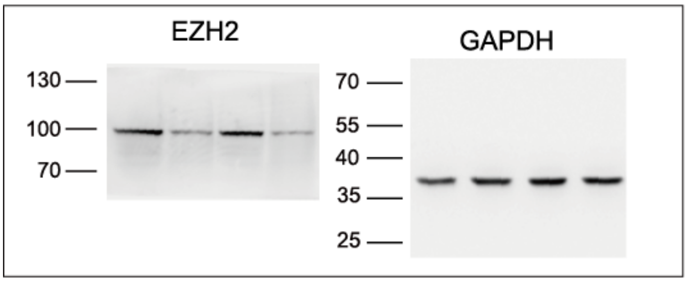

Supplementary Figure 7: Uncropped images of immunoblots (continued).

Supplementary Table 1: Correlation between CXCR4 expression and clinicopathological characteristics of 127 HCCs

Characteristic	Patients(n=127)		CXCR4 expression		
	Number	%	High (n=63)	Low (n=64)	*P* value
Age (years)†	127	100	50.4±11.6	51.3±11.4	0.677
Gender					0.424
Male	109	85.8	52	57	
Female	18	14.2	11	7	
Liver cirrhosis					0.801
Absent	113	89.0	57	56	
Present	14	11.0	6	8	
Child-Pugh					0.203
A	121	95.3	58	63	
B	6	4.7	5	1	
HBsAg					0.106
Negative	15	11.8	4	11	
Positive	112	88.2	59	53	
ALT(U/I)					0.538
≤ 40	58	45.7	31	27	
> 40	69	54.3	32	37	
AFP (ng/ml)					0.273
≤ 20	39	30.7	16	23	
> 20	88	69.3	47	41	
Tumor size					**0.003**
≤ 5	76	59.8	29	47	
> 5	51	40.2	34	17	
Tumor number					0.854
Single	103	81.1	51	52	
Multiple	24	18.9	12	12	
Tumor encapsulation					0.250
Complete	66	52.0	29	37	
None	61	48.0	34	27	
Tumor differentiation					0.953
I-II	88	69.3	43	45	
III-IV	39	30.7	20	19	
Venous invasion					**0.006**
Absent	63	49.6	23	40	
Present	64	50.4	40	24	
BCLC stage					**0.003**
0+A	70	55.1	26	44	
B+C	57	44.9	37	20	
TNM stage					**< 0.001**
I+II	74	58.3	27	47	
III	53	41.7	36	17	

Note: Bold values indicate $P < 0.05$, P values from student's t-test for age, Fisher's exact test for dichotomous variables, or chi-square when taking all categories into account. HBsAg, hepatitis B surface antigen, ALT, alanine aminotransferase, AFP, alpha-fetoprotein, BCLC, the Barcelona Clinic Liver Cancer staging

Supplementary Table 2: Univariate cox regression analyses of overall survival and recurrence free survival after surgery in 127 HCCs

**Characteristics**	**OS**		**RFS**	
	**HR (95% CI)**	***P***	**HR (95% CI)**	***P***
Age	0.996 (0.975-1.018)	0.744	1.000 (0.979-1.022)	0.973
Gender (male *vs* female)	2.108 (0.961-5.037)	0.061	2.112 (0.972-4.186)	0.056
Liver cirrhosis (present *vs* absent)	1.231 (0.532-2.846)	0.629	0.920 (0.441-1.920)	0.826
Child-Pugh (B *vs* A)	2.587 (1.115-6.002)	**0.028**	1.245 (0.455-3.408)	0.672
HBsAg (positive *vs* negative)	1.386 (0.599-3.206)	0.448	0.682 (0.358-1.298)	0.246
ALT, U/I (> 40 *vs* ≤ 40)	1.336 (0.804-2.220)	0.266	1.099 (0.682-1.773)	0.699
AFP, ng/ml (> 20 *vs* ≤ 20)	2.101 (1.119-3.942)	**0.022**	1.112 (0.665-1.860)	0.686
Tumor encapsulation (none *vs* complete)	1.844 (1.112-3.060)	**0.018**	1.806 (1.116-2.923)	**0.017**
Tumor differentiation (III-IV *vs* I-II)	1.673 (0.997-2.805)	0.053	1.456 (0.878-2.414)	0.148
Tumor size, cm (>5 *vs* ≤5)	3.192 (1.870-5.449)	< **0.001**	2.096 (1.279-3.402)	**0.003**
Tumor number (multiple *vs* single)	1.098 (0.597-2.021)	0.764	0.962 (0.516-1.791)	0.903
Vascular invasion (present *vs* absent)	2.743 (1.616-4.655)	< **0.001**	2.114 (1.299-3.442)	**0.003**
BCLC (B+C *vs* 0+A)	4.265 (2.474-7.350)	< **0.001**	2.475 (1.520-4.031)	< **0.001**
TNM (III *vs* I+II)	4.062 (2.390-6.904)	< **0.001**	1.879 (1.162-3.039)	**0.011**
CXCR4 (high *vs* low)	3.605 (2.072-6.272)	< **0.001**	2.194 (1.345-3.580)	**0.002**

Note: Bold values indicate $P < 0.05$. *P* values from cox regression analysis. CI, confidence interval; HR, hazard ratio.

Supplementary Table 3: miRNAs expression in HCC and peritumoral tissues

miRNA	mature sequence	tumor	peritumor	fold change (t/p)
miR-494	UGAAACAUACACGGGAAACCUC	661.5784	525.5734	1.04
miR-185	UGGAGAGAAAGGCAGUUCCUGA	242.3044	178.8209	1.06
miR-204	UUCCCUUUGUCAUCCUAUGCCU	79.3291	66.78498	1.04
miR-224	CAAGUCACUAGUGGUUCCGUU	180.3124	105.5249	1.11
miR-381	UAUACAAGGGCAAGCUCUCUGU	9.389448	50.83247	-1.75
miR-186	CAAAGAAUUCUCCUUUUGGGCU	65.57133	74.00137	-1.03
miR-302d	UAAGUGCUUCCAUGUUUGAGUGU	5.362223	8.033267	-1.24
miR-545	UCAGCAAACAUUUAUUGUGUGC	2.878668	4.283403	-1.38
miR-373	GAAGUGCUUCGAUUUUGGGGUGU	2.338321	4.475798	-1.94
miR-520b	AAAGUGCUUCCUUUUAGAGGG	2.321819	5.450765	-1.96
miR-548e	AAAAACUGAGACUACUUUUGCA	3.797283	4.367013	-1.10
miR-1183	CACUGUAGGUGAUGGUGAGAGUGGGCA	2.777272	6.604022	-1.85
miR-603	CACACACUGCAAUUACUUUUGC	3.64929	6.436064	-1.44
miR-483-3p	UCACUCCUCUCCUCCCGUCUU	14.65592	32.72705	-1.30
Let-7g	UGAGGUAGUAGUUUGUACAGUU	4.312671	4.390575	-1.01
miR-520a-3p	AAAGUGCUUCCCUUUGGACUGU	5.816546	4.543678	1.16
miR-491-3p	CUUAUGCAAGAUUCCCUUCUAC	2.59356	3.263806	-1.24
miR-487b	AAUCGUACAGGGUCAUCCACUU	28.31648	33.02172	-1.05
miR-588	UUGGCCACAAUGGGUUAGAAC	7.131973	4.333252	1.34
miR-586	UAUGCAUUGUAUUUUUAGGUCC	3.510287	4.376949	-1.18
miR-519c-3p	AAAGUGCAUCUUUUUAGAGGAU	4.860589	4.433626	1.06
miR-548b-3p	CAAGAACCUCAGUUGCUUUUGU	3.38019	3.450607	-1.02
miR-329	AACACACCUGGUUAACCUCUUU	2.314246	3.50946	-1.50
miR-570	CGAAAACAGCAAUUACCUUUGC	3.485425	5.43387	-1.36
miR-520c-3p	AAAGUGCUUCCUUUUAGAGGGU	4.923935	8.816524	-1.37
miR-300	AAAGUGCUUCCUUUUAGAGGGU	3.305262	5.835495	-1.48
miR-302b	UAAGUGCUUCCAUGUUUUAGUAG	3.460078	6.438606	-1.50
miR-634	AACCAGCACCCCAACUUUGGAC	4.769967	4.19367	1.09
miR-613	AGGAAUGUUCCUUCUUUGCC	2.189799	2.449947	-1.14
miR-873	GCAGGAACUUGUGAGUCUCCU	2.7184	1.852728	1.62
miR-410	AAUAUAACACAGAUGGCCUGU	6.378645	3.653405	1.43
miR-495	AAACAAACAUGGUGCACUUCUU	6.089215	10.70869	-1.31
miR-520d-3p	AAAGUGCUUCUCUUUGGUGGGU	3.291087	1.75918	2.11
miR-338-3p	UCCAGCAUCAGUGAUUUUGUUG	3.803744	3.225022	1.14
miR-548c-3p	CAAAAAUCUCAAUUACUUUUGC	1.244822	1.3679	-1.43
miR-302e	AAAGCUGGGUUGAGAAGG	1.6257	2.467117	-1.86
miR-548g	AAAACUGUAAUUACUUUUGUAC	2.461921	2.447566	1.01
miR-1299	UUCUGGAAUUCUGUGUGAGGGA	1.414882	1.458405	-1.09
miR-655	AUAAUACAUGGUUAACCUCUUU	3.488609	5.526509	-1.37
miR-623	AUCCCUUGCAGGGGCUGUUGGGU	1.767646	1.54252	1.31

miR-548p	UAGCAAAAACUGCAGUUACUUU	2.630977	3.688518	-1.35
miR-548f	UGCAAAAGUAAUCACAGUUUUU	1.794852	2.275522	-1.41
miR-548d-3p	CAAAAACCACAGUUUCUUUUGC	2.441227	2.441227	-1.00
miR-519a	AAAGUGCAUCCUUUUAGAGUGU	3.048649	1.579421	2.44
miR-153	UUGCAUAGUCACAAAAGUGAUC	2.718713	3.341707	-1.21
miR-1297-3p	UUCAAGUAAUUCAGGUG	2.933135	2.521644	1.16
miR-338-5p	UCCAGCAUCAGUGAUUUUGUUG	1.483307	1.65247	-1.27
miR-659	CUUGGUUCAGGGAGGGUCCCCA	3.79243	2.478187	1.47
miR-448	UUGCAUAUGUAGGAUGUCCCAU	2.526329	2.270782	1.13
miR-302a	UAAGUGCUUCCAUGUUUUGGUGA	3.024506	2.076679	1.51
miR-211	UUCCCUUUGUCAUCCUUCGCCU	7.093168	5.011703	1.22
miR-1284	UCUAUACAGACCCUGGCUUUUC	5.435213	2.521771	1.83
miR-548a-3p	CAAAACUGGCAAUUACUUUUGC	2.537592	2.56968	-1.01
miR-520e	AAAGUGCUUCCUUUUUGAGGG	2.784481	2.924389	-1.05
miR-519b-3p	AAAGUGCAUCCUUUUAGAGGUU	4.470954	6.817912	-1.28
miR-513b	UUCACAAGGAGGUGUCAUUUAU	3.35167	3.032499	1.09
miR-582-5p	UUACAGUUGUUCAACCAGUUACU	3.089191	9.875985	-1.96
miR-372	AAAGUGCUGCGACAUUUGAGCGU	2.164811	4.425403	-1.93
miR-1	UGGAAUGUAAAGAAGUAUGUAU	1.835526	3.202492	-1.92
miR-302c	UAAGUGCUUCCAUGUUUCAGUGG	1.788039	3.54087	-2.18
miR-139-5p	UCUACAGUGCACGUGUCUCCAG	21.06479	1001.924	-2.27
miR-9	UCUUUGGUUAUCUAGCUGUAUGA	3.680397	49.03445	-2.99
miR-206	UGGAAUGUAAGGAAGUGUGUGG	1.752483	6.491815	-3.33
miR-622	ACAGUCUGCUGAGGUUGGAGC	1.241534	4.810622	-7.26

Supplementary Table 4: Target sequences of shRNA

shRNA target	Target sequence	
Luc	GGATTTCAGTCGATGTACACGTTCGTCAC	
CXCR4-1	TTCTTCGCCTGTTGGCTGCCTTACTACAT	TR313630A
CXCR4-2	AGTCAACCTCTACAGCAGTGTCCTCATCC	TR313630B
CXCR4-3	CCATCCTCTATGCTTTCCTTGGAGCCAAA	TR313630C
EZH2-1	CAAGAGGTTCAGACGAGCTGATGAAGTAA	TR304713A
EZH2-2	GACGATGATGATGATGGAGACGATCCTGA	TR304713B
EZH2-3	ATTGAACCTCCTGAGAATGTGGAGTGGAG	TR304713C

Supplementary Table 5: Primers used for gene cloning and qRT-PCR

Name		Sequence
CXCR4 3’UTR	Forward	5’-GCTCTAGACTGTTTCCACTGAGTCTGAG-3’
	Reverse	5’-GCGGCCGGCCACACTGTAGGTGCTGAAATC-3’
CXCR4 3'UTR-Mut	Forward	5’-CATTTTTCAGATATAAATCTGACAC-3’
	Reverse	5’-CAATATTGGTGTCAGATTTATATC-3’
CXCR4	Forward	5’-GGTGGTCTATGTTGGCGTCT-3’
	Reverse	5’-GTCATTGGGGTAGAAGCGGT-3’
GAPDH	Forward	5’-CTGCACCACCAACTGCTTAG-3’
	Reverse	5’-GCCATCCACAGTCTTCTGGG-3’
miR-494	Forward	5’-TGAAACATACACGGGAAACCTC-3’
miR-185	Forward	5’-TGGAGAGAAAGGCAGTTCCTGA-3’
miR-204	Forward	5’-TTCCCTTTGTCATCCTATGCCT-3’
miR-224	Forward	5’-CAAGTCACTAGTGGTTCCGTT-3’
miR-381	Forward	5’-AGCGAGGTTGCCCTTTGTATAT-3’
miR-186	Forward	5’-CAAAGAATTCTCCTTTTGGGCT-3’
miR-302d	Forward	5’- ACTTTAACATGGAGGCACTTGC -3’
miR-545	Forward	5’- TCAGTAAATGTTTATTAGATGA-3’
miR-373	Forward	5’- ACTCAAAATGGGGGCGCTTTCC-3’
miR-520b	Forward	5’- AAAGTGCTTCCTTTTAGAGGG -3’
miR-548e	Forward	5’- CAAAAGCAATCGCGGTTTTTGC -3’
miR-1183	Forward	5’- CACTGTAGGTGATGGTGAGAGTGGGCA-3’
miR-603	Forward	5’- CACACACTGCAATTACTTTTGC-3’
miR-483-3p	Forward	5’- TCACTCCTCTCCTCCCGTCTT-3’
Let-7g	Forward	5’-TGAGGTAGTAGTTTGTACAGTT-3’
miR-520a-3p	Forward	5’- AAAGTGCTTCCCTTTGGACTGT-3’
miR-491-3p	Forward	5’- CTTATGCAAGATTCCCTTCTAC-3’
miR-487b	Forward	5’- GTGGTTATCCCTGTCCTGTTCG -3’
miR-588	Forward	5’- TTGGCCACAATGGGTTAGAAC-3’
miR-586	Forward	5’- TATGCATTGTATTTTTAGGTCC-3’
miR-519c-3p	Forward	5’- AAAGTGCATCTTTTTAGAGGAT-3’
miR-548b-3p	Forward	5’- CAAGAACCTCAGTTGCTTTTGT-3’
miR-329	Forward	5’- GAGGTTTTCTGGGTTTCTGTTTC-3’
miR-570	Forward	5’- AAAGGTAATTGCAGTTTTTCCC-3’
miR-520c-3p	Forward	5’- AAAGTGCTTCCTTTTAGAGGGT-3’
miR-300	Forward	5’- TATACAAGGGCAGACTCTCTCT-3’
miR-302b	Forward	5’- ACTTTAACATGGAAGTGCTTTC-3’
miR-634	Forward	5’- AACCAGCACCCCAACTTTGGAC-3’
miR-613	Forward	5’- AGGAATGTTCCTTCTTTGCC-3’
miR-873	Forward	5’- GCAGGAACTTGTGAGTCTCCT-3’
miR-410	Forward	5’- AGGTTGTCTGTGATGAGTTCG-3’

miR-495	Forward	5'- GAAGTTGCCCATGTTATTTTCG-3'
miR-520d-3p	Forward	5'- AAAGTGCTTCTCTTTGGTGGGT-3'
miR-338-3p	Forward	5'- TCCAGCATCAGTGATTTTGTTG-3'
miR-548c-3p	Forward	5'- CAAAAATCTCAATTACTTTTGC-3'
miR-302c	Forward	5'- TTTAACATGGGGGTACCTGCTG-3'
miR-548g	Forward	5'- TGCAAAAGTAATTGCAGTTTTTG-3'
miR-1299	Forward	5'- TTCTGGAATTCTGTGTGAGGGA-3'
miR-655	Forward	5'- AGAGGTTATCCGTGTTATGTTC-3'
miR-623	Forward	5'- ATCCCTTGCAGGGGCTGTTGGGT-3'
miR-548p	Forward	5'- TAGCAAAAACTGCAGTTACTTT-3'
miR-548f	Forward	5'- TGCAAAAGTAATCACAGTTTTT-3'
miR-548d-3p	Forward	5'- CAAAAACCACAGTTTCTTTTGC-3'
miR-519a	Forward	5'- CTCTAGAGGGAAGCGCTTTCTG-3'
miR-153	Forward	5'- TCATTTTTGTGATGTTGCAGCT-3'
miR-1207-3p	Forward	5'- TCAGCTGGCCCTCATTTC-3'
miR-338-5p	Forward	5'- AACAATATCCTGGTGCTGAGTG-3'
miR-659	Forward	5'- AGGACCTTCCCTGAACCAAGGA-3'
miR-448	Forward	5'- TTGCATATGTAGGATGTCCCAT-3'
miR-302a	Forward	5'- ACTTAAACGTGGATGTACTTGCT-3'
miR-211	Forward	5'- TTCCCTTTGTCATCCTTCGCCT-3'
miR-1284	Forward	5'- TCTATACAGACCCTGGCTTTTC-3'
miR-548a-3p	Forward	5'- CAAAACTGGCAATTACTTTTGC-3'
miR-520e	Forward	5'- AAAGTGCTTCCTTTTTGAGGG-3'
miR-519b-3p	Forward	5'- AAAGTGCATCCTTTTAGAGGTT-3'
miR-513b	Forward	5'- TTCACAAGGAGGTGTCATTTAT-3'
miR-582-5p	Forward	5'- TTACAGTTGTTCAACCAGTTACT-3'
miR-372	Forward	5'- CCTCAAATGTGGAGCACTATTCT-3'
miR-9	Forward	5' TCTTTGGTTATCTAGCTGTATGA--3'
miR-302c	Forward	5'- TTTAACATGGGGGTACCTGCTG-3'
miR-139-5p	Forward	5'- TCTACAGTGCACGTGTCTCCAGT-3'
miR-1	Forward	5'- TGGAATGTAAAGAAGTATGTAT-3'
miR-206	Forward	5'- TGGAATGTAAGGAAGTGTGTGG-3'
miR-622	Forward	5'- ACAGTCTGCTGAGGTTGGAGC-3'
U6snRNA	Forward	5'-CTCGCTTCGGCAGCACA-3'
	Reverse	5'-AACGCTTCACGAATTTGCGT-3'
Forward primers for mature miRNAs were provided here, reverse primer was provided by Invitrogen as Universal qRT-PCR primer.		

Supplementary Table 6: All primary antibodies against the indicted proteins used in the present study are listed

Antibody	Provider	Host	Dilution
CXCR4	Millipore (AB1846)	Rabbit	1:1000 WB<br>1:200 IHC
EZH2	Millipore (07-689)	Rabbit	1 : 1000 WB<br>1:100 CHIP
EZH2	Abcam (ab 3748)	Rabbit	1:400 IHC
H3k27me3	Millipore (17-622)	Rabbit	1: 2000 WB<br>1:100 CHIP
H3K9me3	Millipore (05-745R)	Rabbit	1:2000 WB<br>1:100 CHIP
Histone 3	Millipore (06-755)	Rabbit	1:3000 WB
GAPDH	Santa cruz (sc-365062)	Mouse	1:2000 WB
Lamin B1	Santa cruz (sc-377001)	Mouse	1:4000 WB
Ki 67	DAKO (MIB-1)	Mouse	1:1000 IHC
